# Repeated Origin and Loss of Adhesive Toepads in Geckos

**DOI:** 10.1371/journal.pone.0039429

**Published:** 2012-06-27

**Authors:** Tony Gamble, Eli Greenbaum, Todd R. Jackman, Anthony P. Russell, Aaron M. Bauer

**Affiliations:** 1 Department of Genetics, Cell Biology and Development, University of Minnesota, Minneapolis, Minnesota, United States of America; 2 Bell Museum of Natural History, University of Minnesota, St. Paul, Minnesota, United States of America; 3 Department of Biology, Villanova University, Villanova, Pennsylvania, United States of America; 4 Department of Biological Sciences, University Department of Calgary, Calgary, Canada; Institute of Evolutionary Biology (CSIC-UPF), Spain

## Abstract

Geckos are well known for their extraordinary clinging abilities and many species easily scale vertical or even inverted surfaces. This ability is enabled by a complex digital adhesive mechanism (adhesive toepads) that employs van der Waals based adhesion, augmented by frictional forces. Numerous morphological traits and behaviors have evolved to facilitate deployment of the adhesive mechanism, maximize adhesive force and enable release from the substrate. The complex digital morphologies that result allow geckos to interact with their environment in a novel fashion quite differently from most other lizards. Details of toepad morphology suggest multiple gains and losses of the adhesive mechanism, but lack of a comprehensive phylogeny has hindered efforts to determine how frequently adhesive toepads have been gained and lost. Here we present a multigene phylogeny of geckos, including 107 of 118 recognized genera, and determine that adhesive toepads have been gained and lost multiple times, and remarkably, with approximately equal frequency. The most likely hypothesis suggests that adhesive toepads evolved 11 times and were lost nine times. The overall external morphology of the toepad is strikingly similar in many lineages in which it is independently derived, but lineage-specific differences are evident, particularly regarding internal anatomy, with unique morphological patterns defining each independent derivation.

## Introduction

Repeated evolution, also called convergent or parallel evolution, is the independent emergence of similar traits in separate evolutionary lineages and is typically seen as evidence of adaptation through natural selection or of developmental constraints that limit or bias morphological evolution [1], [2], [3], [4], [5]. Examining instances of repeated evolution serves as an important means of studying evolutionary processes and is analogous to studying multiple experimental replicates [6]. Indeed, each case of convergent or parallel evolution reveals the degree of common response to some fundamental biological challenge. As a result, extensive effort has been devoted to identifying instances of repeated evolution. To do this effectively, an accurate phylogeny is required for the “mapping” of traits and to permit examination of whether similarity is the result of shared ancestry or represents true independent derivation [3]. Many aspects of vertebrate body form related to locomotion have evolved repeatedly, being both gained and lost many times over. This includes functionally significant traits such as wings as aerodynamic devices, and limb reduction or elimination associated with burrowing [7], [8], [9]. Likewise, adhesive toepads employed in climbing have evolved several times in vertebrates, including multiple lineages of treefrogs, *Anolis* lizards, *Prasinohaema* skinks and, perhaps most notably in geckos [10], [11].

The key component of the adhesive apparatus in lizards is the presence of setae, microscopic hair-like outgrowths of the superficial layer of the subdigital epidermis (the Oberhäutchen), which promote adhesion via van der Waals forces and complex frictional interactions [12], [13], [14], [15]. Setae evolved from the microscopic spinules that are typical of the outer epidermis of all limbed gekkotans and some other squamates [15], [16], [17], [18], and are hypothesized to aid in skin shedding [16], [19]. A hierarchy of anatomical specializations have evolved to govern the adhesive properties of the setae, and dynamic interactions with the substrate depend on numerous morphological adaptations and behaviors that facilitate control of the adhesive mechanism during locomotion [13], [20], [21], [22], [23]. Collectively, these specializations permit effective and rapid application and removal of the setae with reference to the substrate and constitute a functionally integrated complex [13], [24].

Geckos are among the most species-rich and geographically widespread of terrestrial vertebrate lineages, with ∼1450 described species in 118 genera, and comprise 25% of all described lizard species [25]. They are the likely sister group of all other lizards and snakes, excluding the limbless dibamids, having diverged from other squamates 225–180 MY ago [26], [27]. The gekkotan adhesive system has been present since at least the mid-Cretaceous, as revealed by scansorial pads preserved in amber-embedded gecko fossils [28], [29]. Approximately 60% of gecko species possess adhesive toepads, whereas the remainder lack functional adhesive toepads (or lack limbs altogether, in the case of the Australian pygopodid geckos) [7]. Geckos with adhesive toepads can easily scale vertical or even inverted surfaces, and these extraordinary clinging abilities have long attracted scientific attention [16], [30], [31]. Recently, interest has focused on mimicking the gecko adhesive mechanism to develop bio-inspired technologies [32], [33], [34]. Biomimetic studies have concentrated largely on adhesion at the molecular level, but functional control of adhesive toepads requires integration across a hierarchy of systems operating at different scales. These complex interactions – from molecular bonds to the locomotor control of the entire organism – are incorporated across seven orders of magnitude of size in geckos [13].

The form and structure of adhesive toepads in geckos have been used historically for taxonomic purposes, chiefly for assigning species to genera [35], [36], [37]. Traditional views of gecko evolution presupposed a single [38], or at most two [22], origins of the adhesive apparatus. These views were inferred from phylogenetic hypotheses that used few characters and sparse taxon sampling, and that placed the padless eublepharid geckos as sister to all remaining geckos, a position refuted by recent molecular phylogenies [26], [27], [39], [40]. Reconstructing the evolution of gekkotan adhesive toepads, therefore, requires a comprehensive phylogeny derived from an independent data source, i.e., molecular genetic data. Here we estimate the phylogenetic relationships among nearly all recognized gecko genera using a multilocus dataset. We optimize the evolution of adhesive toepads on this phylogeny and reveal extensive homoplasy both in toepad morphology and in patterns of toepad loss. Our approach provides an appropriate framework for investigating broader functional and ecological questions that are associated with the origin, diversification and secondary loss of adhesive toepads. Being able to focus upon evolutionary events in different parts of the gekkotan phylogeny will permit more specific questions to be explored. In this contribution we provide exemplars of such phenomena, and consider the environmental circumstances that may have triggered particular transitions. Further explorations of similar transitions in other parts of the phylogeny will ultimately lead to potential generalizations about the form, function and adaptive significance of adhesive pad configuration in its various guises.

## Methods

### Phylogenetic Analyses

We estimated phylogenies using approximately 4,100 aligned bases of nucleotide data, from 244 gekkotan taxa and 14 outgroups (Table S1). The dataset was mostly complete, with only about 3% missing data. This included exemplars from 107 of 118 recognized gekkotan genera. Several recently described or elevated genera [41], [42] were not sampled, but these new taxa are invariant in digital morphology in comparison to related taxa that are represented in our phylogenetic analyses. DNA sequence data consisted of fragments of five nuclear protein-coding genes: *RAG1*, *RAG2*, *C–MOS*, *ACM4*, and *PDC*; and one mitochondrial gene: *ND2* and associated tRNAs. Primers, PCR conditions, and sequencing conditions are detailed elsewhere [43], [44]. Sequence data have been deposited in GenBank (Table S1). We aligned sequences using T-Coffee [45] with default parameters and fine-tuned alignments by hand to ensure insertions and deletions did not disrupt the translation of DNA sequence into amino acids. Protein-coding sequences were translated into amino acids using MacClade 4.08 [46] to confirm alignment and gap placement. Alignment gaps were treated as missing data and nuclear gene sequences were unphased. We estimated phylogenetic relationships among taxa using Maximum Likelihood (ML) in RAxML 7.2.6 [47] and Bayesian analysis in MrBayes 3.1.2 [48]. Data in both analyses were divided into seven partitions; first by genome (nDNA and mtDNA) and then by codon, with a separate partition for tRNAs. This partitioning scheme contains fewer parameters than the preferred partitioning strategy used in previous phylogenetic analyses of the same nuclear loci (partitioning by both gene and codon), but with far fewer taxa [43], [49]. The more parameter-rich strategy resulted in convergence problems in the Bayesian analysis of this taxon-rich dataset, likely due to low phylogenetic signal in the smaller partitions; these problems were resolved by reducing the number of partitions. Model selection was based on AIC scores using the software jModeltest [50], which recovered either the GTR + *I* + *G* or the GTR + *G* models for each partition (Table S2). The GTR + *G* model was used for all partitions in the ML analysis, which is the only model implemented in RAxML due to problematic interactions between the *I* and *G* parameters [51], [52]. Bayesian analyses were run with multiple MCMC chains for 40 million generations, sampling every 1000^th^ generation. Post burn-in convergence was checked by visual inspection of likelihood values by generation using Tracer 1.5 [53] and comparing split frequencies between runs using AWTY [54].

### Comparative Analyses

We categorized digital morphologies in all sampled taxa as a binary character, coding species lacking a functional digital adhesive mechanism as 0 and species with a functional digital adhesive mechanism as 1 (Table S1). Morphological data were gathered from the literature as well as our personal examination of museum specimens representing 95% of described gecko species. Methods summarizing the collection of paraphalangeal data have been detailed elsewhere [21].

We estimated the number of independent gains and losses of the gekkotan digital adhesive mechanism using ancestral state reconstruction under parsimony and Maximum Likelihood in Mesquite [55], and Bayesian reconstruction in Bayestraits [56]. We incorporated phylogenetic uncertainty into our ancestral state reconstructions by summarizing ancestral states over a random subsample of 5,000 post burn-in trees from the Bayesian phylogenetic analyses onto the ML tree [57]. To investigate whether gains and losses of a functional digital adhesive mechanism occurred at the same rate in geckos, we compared the 1–rate MK1 model [58] to the asymmetric 2–rate model [59], [60] with the likelihood ratio test in both the ML and Bayesian reconstructions.

Ancestral state reconstruction methods can be positively misleading if the trait in question influences diversification rates [61], [62]. To correct for this artefact we used the binary-state speciation and extinction (BiSSE) model [61] to simultaneously estimate transition rates between binary characters (q01 and q10) and state-specific extinction (mu0 and mu1) and speciation rates (lambda0 and lambda1). We accounted for the incomplete species sampling of our phylogeny (∼10% of described gekkotan species) by converting our ML phylogeny into a terminally-unresolved generic-level tree that could accommodate all unsampled taxa [63]. We pruned our phylogeny to 107 terminal taxa, roughly equivalent to genera, to which we could unambiguously assign all 1,452 described gecko species. There were several instances where multiple genera were grouped together for convenience, as well as several instances where genera were split into multiple groups due to the revelation of generic paraphyly (see results). In all cases, there were no changes in the presence or absence of adhesive toepads among impacted clades, so any influence of this taxonomic assignment on our results should be negligible. The ML phylogeny was made ultrametric using penalized likelihood in APE 2.7 [64], [65] with the root arbitrarily scaled to 100. We calculated BiSSE model parameters from the ultrametric ML tree using maximum likelihood in the software Diversitree [63]. We also tested several hypotheses regarding the evolution of the digital adhesive mechanism using a range of constrained BiSSE models. We calculated parameters for the unconstrained, six-parameter model and then sequentially constrained each of the model parameters, alone and in combination, to yield a single rate for each parameter (e.g., mu0  =  mu1, lambda0  =  lambda1, q01  =  q10) to determine if constrained models provided a better fit to the data than did the unconstrained model. We also explored whether models that restricted transitions between character states provided a realistic evaluation of our data. We did this by constraining q01  =  0, where a functional digital adhesive mechanism evolved just once; and q10  =  0, where once gained, a functional digital adhesive mechanism is never lost. We used AIC scores to determine which model provided the best fit to our data. Bayesian posterior distributions of BiSSE model parameters were also estimated using Markov Chain Monte Carlo analyses with the terminally unresolved generic-level ML tree in Diversitree [63]. Priors for each parameter used an exponential distribution, and estimated ML model parameters were used as a starting point. We combined results from two separate MCMC chains run for 10,000 generations each, with the first 10% of each run discarded as burn-in.

## Results

**Figure 1:**
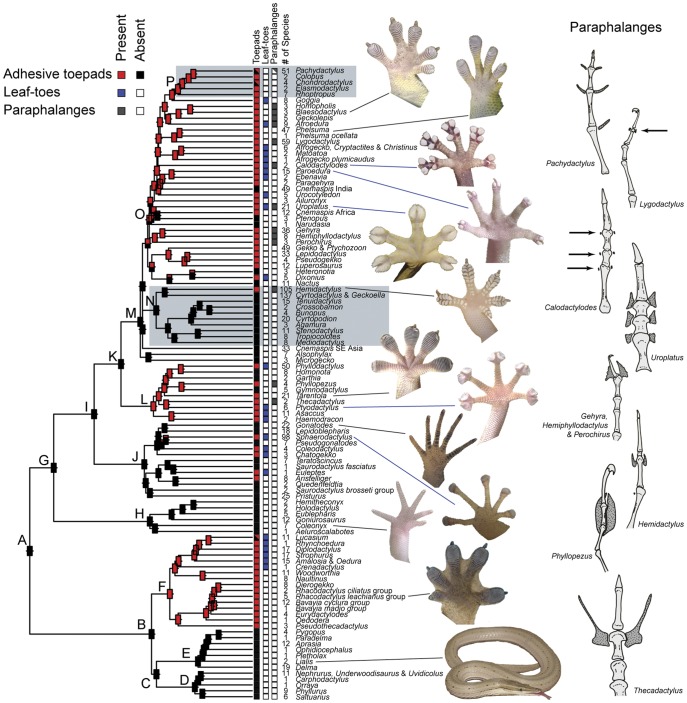
Gecko phylogeny and the evolution of adhesive toepads. Maximum likelihood tree showing phylogenetic relationships among gecko genera. Toepad traits, including the presence of adhesive toepads, toepad shape and the presence of paraphalanges, are illustrated by colored squares on the tips of the branches (squares with two colors indicate polymorphism within the clade). Rectangles at internal nodes represent ancestral presence or absence probabilities of adhesive toepads inferred using the 6-parameter binary-state speciation and extinction (BiSSE) model. Details for lettered clades are presented in Table 1. Representative images illustrate a variety of gecko toepad morphologies. Single digits from representative gecko species illustrating the morphological diversity of paraphalangeal elements (in gray with stippling) are shown on the right. Clades enclosed in gray boxes are shown in greater detail in Figures 3 and 4.

Molecular phylogenies recover patterns of interfamilial relationships consistent with previous molecular studies (Fig. 1, Figs. S1-S2) [26], [39], [40], [43]. This includes well-supported monophyly of all seven gekkotan families (Table 1, Figs. S1-S2), with both Bayesian and maximum-likelihood trees concordant at well-supported nodes. Portions of the phylogeny with short internal branches are generally poorly supported, making it difficult to resolve phylogenetic relationships among many genera. This is the case at the base of Gekkonidae, Phyllodactylidae and Sphaerodactylidae. Several recognized genera are recovered with strong support as either para- or polyphyletic: *Afrogecko, Cnemaspis, Cyrtodactylus, Gekko, Rhacodactylus* and *Saurodactylus*.

**Table 1:** Nodal support and ancestral states for key nodes of the gecko phylogeny.

Node	Clade Name	P(toepads)	ML bootstrap	Bayesian PP	Age (mya)
A	Gekkota	0.014 (0.000–0.035)	100	1.00	118–167
B	Pygopodoidea	0.233 (0.063–0.386)	100	1.00	66–102
C	unnamed	0.034 (0.000–0.136)	52	0.71	59–95
D	Carphodactylidae	0.000 (0.000–0.002)	100	1.00	20–46
E	Pygopodidae	0.000 (0.000–0.000)	100	1.00	28–44
F	Diplodactylidae	0.999 (0.999–1.00)	100	1.00	47–78
G	Gekkomorpha	0.020 (0.001–0.005)	92	1.00	113–157
H	Eublepharidae	0.001 (0.000–0.002)	100	1.00	60–98
I	Gekkonoidea	0.194 (0.031–0.386)	100	1.00	96–132
J	Sphaerodactylidae	0.008 (0.001–0.017)	100	1.00	85–117
K	unnamed	0.908 (0.775–0.997)	100	1.00	82–114
L	Phyllodactylidae	0.999 (0.998–1.00)	100	1.00	63–93
M	Gekkonidae	0.205 (0.008–0.523)	100	1.00	73–101
N	unnamed	0.020 (0.008–0.034)	100	1.00	60–87
O	Afro–Malagasy Clade	0.994 (0.973–1.00)	22	0.99	73–100
P	*Pachydactylus* Clade	0.998 (0.995–0.999)	100	1.00	41–69

Node labels refer to Figure 1. Posterior probabilities of the presence of toepads, *P*(toepads), calculated from the Bayesian comparative analysis. Nodal support values include maximum likelihood bootstrap values and Bayesian posterior probabilities. Node ages are from [39].

**Figure 2:**
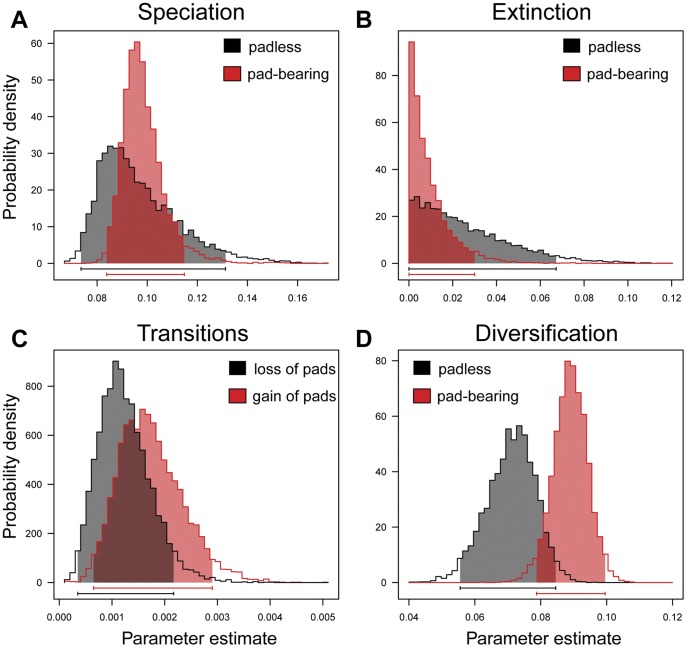
Bayesian parameter estimates inferred using the 6-parameter binary-state speciation and extinction (BiSSE) model. Estimates of: A. trait-specific speciation rates (lambda); B. trait-specific extinction rates (mu); C. transition rate parameters (q01 =  gain of adhesive toepads, q10 =  loss of adhesive toepads); D. net diversification rates calculated as the difference between speciation (lambda) and extinction (mu) rates for genera with and without adhesive toepads. The 95% credibility intervals for each parameter are shaded and indicated by bars along the x-axis.

Comparative analyses using multiple methodologies reveal repeated gains and losses of adhesive toepads (Fig. 1, Figs. S3, S4, S5 and S6). Phylogenetic uncertainty, due to short internodes, makes unambiguous ancestral state reconstructions difficult in some parts of the tree, particularly within the Gekkonidae (Fig. S4). Even so, well-resolved, strongly supported nodes across the phylogeny provide clear evidence of independent gains and losses. Reconstructing ancestral character states with parsimony (Fig. S5) across a selection of trees from the Bayesian phylogenetic analysis results in 20 transitions, with an average of 11 gains (min = 3, max = 17) and 9 losses (min = 3, max = 18). Indeed, gains and losses occur at about the same rate in all of our analyses (Fig. S6). A 1–rate transition model yields results that are not significantly different from an asymmetric 2–rate model for both maximum likelihood reconstructions (likelihood ratio test; *P* = 0.4394) and Bayesian reconstructions (Fig. S3). Similarly, the distribution of character transition rates shows considerable overlap in credibility intervals using a Bayesian implementation of the BiSSE model (Fig. 2) [61], [63]. This extends to overlapping diversification rates (calculated as trait-specific speciation - extinction) among padded and padless lineages (Fig. 2). Comparing the full and constrained maximum likelihood BiSSE models (Table 2) reveals that constraints five and six best fit the data, although AIC differences among most models are small. Constraints five and six both have equal transition rates (q01  =  q10) and constrain either equal speciation rates (constraint 5, lambda0  =  lambda1) or equal extinction rates (constraint 6, mu0  =  mu1). Models that restrict transitions between character states (i.e., constraints eight and nine where q01  =  0, q10  =  0), provide a significantly worse fit to the data than the unconstrained and remaining constrained models. All of the comparative analyses indicate that the most recent common ancestor of all geckos lacked adhesive pads. Many padless lineages retain this ancestral state (e.g., Carphodactylidae and Eublepharidae), but in many others this condition is secondarily derived (e.g., *Homonota*, *Garthia* and *Gymnodactylus*).

**Table 2:** Comparison of full and constrained maximum likelihood binary-state speciation and extinction (BiSSE) models.

Model	constraints	lambda0	lambda1	mu0	mu1	q01	q10	parameters	lnLik	AIC
full	None	0.0919287	0.0916504	0.0196976	0.0000042	0.0015639	0.0011354	6	−775.51	1563.0
constraint 1	lambda0 = lambda1	0.0917114	0.0917114	0.0195089	0.0000005	0.0015538	0.0011399	5	−775.51	1561.0
constraint 2	mu0 = mu1	0.0782689	0.0915912	0.0000147	0.0000147	0.0015438	0.0011227	5	−775.59	1561.2
constraint 3	q01 = q10	0.0917787	0.0917094	0.0197393	0.0000002	0.0013342	0.0013342	5	−775.61	1561.2
constraint 4	lambda0 = lambda1, mu0 = mu1	0.0858881	0.0858881	0.0000149	0.0000149	0.0015973	0.0010989	4	−777.70	1563.4
constraint 5	lambda0 = lambda1, q01 = q10	0.0917360	0.0917360	0.0197087	0.0000034	0.0013375	0.0013375	4	−775.61	**1559.2**
constraint 6	mu0 = mu1, q01 = q10	0.0781298	0.0916917	0.0000117	0.0000117	0.0013121	0.0013121	4	−775.75	**1559.5**
constraint 7	lambda0 = lambda1, mu0 = mu1, q01 = q10	0.0857970	0.0857970	0.0000003	0.0000003	0.0013039	0.0013039	3	−777.92	1561.8
constraint 8	q01 = 0	0.0815919	0.1616154	0.0000076	0.0960942	0.0000000	0.0019102	5	−778.31	1566.6
constraint 9	q10 = 0	0.1898625	0.0943962	0.1408593	0.0000000	0.0036345	0.0000000	5	−820.50	1651.0

Trait 0 lacks adhesive toepads; trait 1 possesses adhesive toepads. Lambda  =  trait specific speciation rates; mu  =  trait specific extinction rates; q  =  transition rate parameters. Constrained models are compared using the Akaike Information Criterion (AIC). The models with the lowest AIC scores are in bold.

## Discussion

Phylogenetic comparative analyses recover multiple gains and losses of adhesive toepads in geckos. This contrasts with previous hypotheses that suggest one, or at most two origins of toepads in geckos [22], [38]. This rampant convergence and parallelism in digital design helps explain the generally poor performance of superficial digital characters for systematic purposes, particularly at higher levels of inclusiveness [22], [43], [66], [67]. Morphological evidence for gekkotan relationships exists, but a high noise-to-signal ratio among the relatively few morphological characters that have been exploited in gecko systematics to date has hampered both phylogenetic reconstruction and the study of character evolution. Recent work using molecular systematic approaches reveals that many gecko genera, originally defined by toepad morphology, are polyphyletic [39], [68], [69], [70]. Here we identify three more polyphyletic genera: *Afrogecko, Cnemaspis* and *Rhacodactylus*. The genera *Gekko* and *Cyrtodactylus* are rendered paraphyletic by *Ptychozoon* and *Geckoella*, respectively. These results indicate that additional work at the generic level is necessary to ensure that gecko taxonomy is isomorphic with phylogeny.

The BiSSE model co-estimates character transition rates and trait-specific speciation and extinction rates, which allows for the estimation of diversification rates (speciation - extinction, Fig. 2) for lineages with and without adhesive toepads. Whereas diversification rates in gecko lineages with toepads are higher than in lineages lacking toepads, these differences are small, and there is overlap in the Bayesian posterior distributions of BiSSE diversification parameters. Therefore, the presence of adhesive toepads, on its own does not appear to have directly influenced the number of species in different gecko lineages. The lack of a direct relationship between adhesive toepads and diversification rates in geckos highlights the complicated relationship between the evolution of complex traits, speciation and extinction. The success of geckos has been linked to possessing many derived traits including nocturnality, visual and olfactory prey discrimination, and shifts in diet, as well as adhesive toepads [71], [72], [73]. That adhesive toepads do not, on their own, explain gecko diversification rates should therefore come as no surprise. Uncovering the patterns and processes that explain the great diversity of geckos overall, as well as the disparities in species richness among gekkotan clades, is a rich source for further research that will be greatly facilitated by the comprehensive phylogeny presented here.

**Figure 3:**
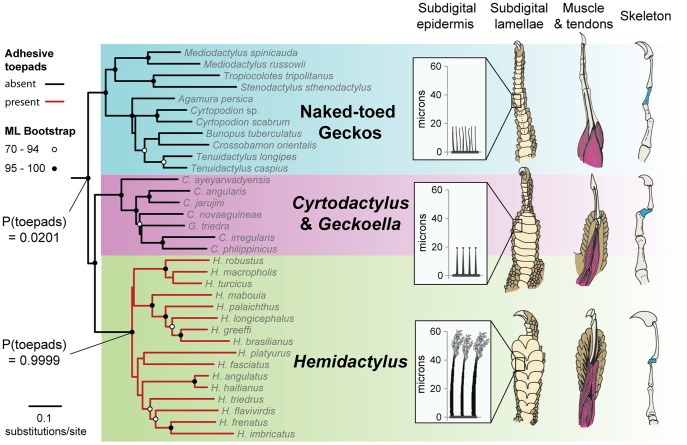
An unambiguous gain of adhesive toepads in house geckos (*Hemidactylus*). Maximum likelihood tree of included *Hemidactylus* species and their close relatives, the padless “naked-toed” geckos and the *Cyrtodactylus* + *Geckoella* clade. Circles at nodes indicate bootstrap support. Bayesian posterior probabilities of the presence of toepads are shown for two key nodes. Selected morphological components that comprise the digital adhesive mechanism are illustrated for each major clade. All three clades share spinules on the subdigital epidermis although only in *Hemidactylus* are they fully elaborated as setae. In the *Cyrtodactylus* + *Hemidactylus* clade: the subdigital lamellae are broadened; the antepenultimate phalanx of the digit (in blue) is reduced and, together with the penultimate phalanx and the claw, forms a raised arc; and the dorsal (extensor) musculature is expanded distally along the digit. The transition to fully functional toepads occurs in *Hemidactylus*, which incorporate the tendinous system that controls individual scansors, and possesses epdidermal spinules that are of increased length and that are multi-spatulate, enhancing functional adhesive surface area. These are recognizable as setae.

An unambiguous gain of adhesive toepads from a padless ancestor is exemplified by the globally distributed genus *Hemidactylus.* The modular construction of the adhesive mechanism is evident when detailed digital morphology is compared to that of related padless genera, and when comparing the elaboration of specialized components from unspecialized precursors (Fig. 3). The likely key initial modification of the digit in *Hemidactylus*, indeed the minimum requirement necessary to possess a functional adhesive mechanism, involves the elaboration of the subdigital spinules into setae with multi-spatulate tips. Because a spinulate epidermis seems ubiquitous among limbed geckos [15], [16], [17], [18], a setal precursor does not need to evolve *de novo* each time the adhesive mechanism evolves. Elongation of the epidermal spinules, initially likely involved in the enhancement of traction [74], influenced the ability of the integumentary outgrowths to interact with the substrate *via* van der Waals forces, promoting further setal elaboration and the subsequent integration of associated morphological traits that control the elaborated setae as a directional adhesive complex [22]. These associated morphological traits in *Hemidactylus*, and indeed all padded gecko lineages, include a broadened subdigital surface (scansors), and modified tendons and muscles to control these scansors. Other modifications specific to *Hemidactylus*, and a few other padded lineages, include a raised penultimate phalanx resulting in a claw that is free of the expanded pad, and neomorphic skeletal structures, the paraphalanges, which aid in the support of the scansors.

**Figure 4:**
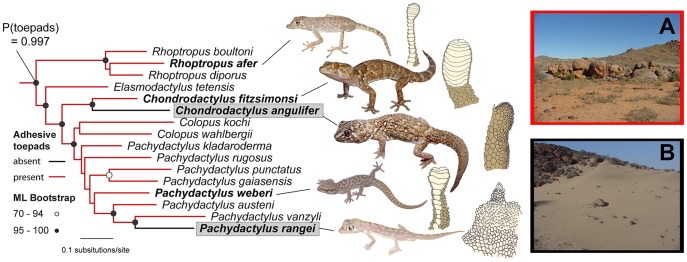
Two unambiguous losses of adhesive toepads in south African geckos. Maximum likelihood tree illustrating two independent losses of the digital adhesive mechanism in the southern African geckos *Chondrodactylus angulifer* and *Pachydactylus rangei* (in shaded boxes). Circles at nodes indicate bootstrap support. Bayesian posterior probabilities of the presence of toepads are shown for the most recent common ancestor of the included lineages, clearly indicating that the ancestor of this group possessed toepads. Representative species and their associated digital morphologies are illustrated. (**A**) Rupicolous habitat where padded members of this clade typically occur. (**B**) Sand dune habitat where the padless *Chondrodactylus angulifer* and the web-footed *Pachydactylus rangei* typically occur.

Adhesive toepads were lost nearly as many times as they originated, and a padless morphology is secondarily derived in many lineages. Unequivocal losses occurred in several lineages of Phyllodactylidae, within the diplodactylid genus *Lucasium* and within the gekkonid genera *Pachydactylus* and *Chondrodactylus* (Fig. 4). The latter three losses are associated with habitat shifts away from a rupicolous lifestyle to burrowing in loose sand [75], and highlight the adaptive significance of toepad morphology. The padless *Chondrodactylus angulifer*, for example, still retains skeletal, muscular and tendinous structures in the digits similar to those of related species that possess a functional adhesive mechanism [76]. The secondary loss of adhesive toepads results in a more highly derived morphology and, consistent with Dollo’s law [77], does not simply reverse to the ostensibly primitive state. This pattern of reduction demonstrates that the adhesive system, once fully assembled, becomes reduced as a functionally integrated structural module [78], [79] that remains fully intact but diminished in size, rather than displaying disassembly and dissolution. This pattern can be seen in six additional species in the genera *Rhoptropus* and *Pachydactylus* that have independently transitioned to terrestriality and show reductions (but not complete loss as seen in *C. angulifer* and *P. rangei*) in the number of scansors and in setal length [75], [80].

Geckos show many lineage-specific morphological traits associated with the repeated gains and losses of adhesive toepads. These traits (which include modifications of the integument, digital skeleton, paraphalanges, musculo-tendinous system, and the vascular sinus network.), when re-examined in light of the hypothesis presented here, allow us to distinguish among most gecko lineages with independently derived adhesive systems as well as identify primitively padless lineages [13], [21], [22], [76]. Two morphological traits associated with the digital adhesive mechanism show multiple independent origins and highlight lineage-specific differences among geckos with adhesive toepads. The first trait is toepad form. Toepads have traditionally been classified either as “leaf-toed,” having divided, expanded scansors at the distal end of the digit, or “basal,” having scansors distributed either proximally or along the entire length of the digit [22]. The leaf-toed morphology evolved in parallel 13–15 times and occurs in all of the major pad-bearing lineages (Fig. 1). Some leaf-toed lineages are independent derivations from a padless ancestor (e.g., *Euleptes*), whereas others are derived from a pad-bearing ancestor with close relatives having basal pads, implying that transitions between pad types are possible (e.g., *Goggia;* the Australian diplodactylids – *Crenadactylus, Oedura, Strophurus, Rhynchoedura, Diplodactylus* and *Lucasium*). Thus, the leaf-toed morphology has originated more often than adhesive pads as a whole, indicating the prevalence of transitions between pad types. The second trait is paraphalanges, cartilaginous or bony neomorphic structures associated with interphalangeal joints and thought to aid in support of the digital scansors or interdigital webbing [21], [75]. Paraphalanges evolved nine times independently in geckos (Fig. 1). In almost every case their morphology is unique and easily distinguishable from those derived in other lineages. Paraphalanges exemplify complex characters that, when interpreted in a morphologically naïve context (e.g., a single binary character), may be seen as highly homoplastic, but if considered in light of specific structure and function (Fig. 1), reveal that each instance is unique.

The repeated gains and losses of the digital adhesive mechanism illustrate the importance of digital morphology in substrate interactions. Adhesive toepads enable animals that posses them to exploit vertically structured habitats, thereby allowing enhanced partitioning of the spatial niche [71], [72]. The ability to adapt to specific substrates, for both digits with and without adhesive toepads, is also an important characteristic of geckos, and regions typified by geologic and topographic heterogeneity have been linked to increased diversity of gecko species [81]. Further research into the gekkotan adhesive mechanism should provide extensive material conducive to the study of the evolution of adaptive, complex phenotypes and partitioning of the spatial niche. Results presented here will prove useful in fostering additional research by identifying lineages with uniquely derived adhesive toepad morphology, and in differentiating between ancestrally padless lineages and species that are secondarily padless. The repeated evolution of adhesive toepads in the diverse and ancient geckos therefore, like the well-studied Caribbean *Anolis* ecomorphs [82], provides an outstanding resource for the understanding of mechanisms that drive phenotypic evolution, the balance between predictable evolutionary outcomes and historical contingency, and the relative influence of adaptation and developmental constraint on convergent and parallel evolution [2], [3], [5], [83]. The sorts of questions that might arise from these considerations relate to particular regions of the phylogeny, rather than to the synthetic bigger picture. Our broad-scale approach characterized adhesive toepads as essentially being present or absent. It does not explore, except for the exemplar taxa chosen, any of the variations in expression of the anatomical components [13], [76] of the adhesive system. Aspects such as the significance of adhesive pad size [84] within and between gekkotan lineages, the manifestation of particular morphological patterns [22], [76] or the environmental circumstances associated with the reduction or loss of the adhesive system [80] necessitate a finer scale of focus. For example, the relative size and configuration of adhesive toepads within lineages requires detailed examination at the species level in association with study, at the microscopic scale, of the locomotor surfaces that they exploit. Such approaches have been conducted for a limited number of taxa [85], [86], and can now be expanded to other parts of the phylogeny to test for congruence in observed patterns. Likewise, localized radiations within the phylogeny can be explored for circumstances related to adhesive pad reduction and loss. Increasing aridity and the exploitation of terrestrial habitats have been associated with such trends in southern Africa and the interior of Australia [75], [76], [87], [88]. Additionally, the evolution of adhesive pad form (leaf-toed versus basal toepad patterns) can now be investigated in detail by pinpointing instances in the phylogeny in which each pattern has arisen independently, and in which transitions from leaf-toed to basal toepad expression have occurred [76], enabling questions about functional and mechanical effectiveness to be investigated.

The diversity of adhesive toepads in geckos holds enormous potential for biomimicry research, not only at the molecular level but also across the entire range of size scales at which geckos operate [12]. Repeated evolution of adhesive toepads can provide the foundation for understanding what is necessary and sufficient to make the “natural” adhesive system operable and functional. That foundation will allow the phylogenetic variation to be stripped away so that basic assembly rules can be understood, which will make formulation of biomimetic approaches more logical. Rather than selecting one exemplar gecko to copy, identifying distinct morphological modules from an array of separate evolutionary origins will permit a simpler and more directed approach to understanding how this functionally integrated complex operates.

## Supporting Information

Figure S1 **Phylogenetic relationships among sampled gecko species estimated using partitioned maximum likelihood.** Bootstrap values from 100 rapid bootstrap replicates are shown at nodes.
(PDF)

Figure S2 **Phylogenetic relationships among sampled gecko species estimated using partitioned Bayesian analysis.** Bayesian posterior probabilities are shown at nodes.
(PDF)

Figure S3 **Gecko phylogeny and the evolution of adhesive toepads estimated using Bayesian methods.** A. Bayesian posterior distributions of the presence of toepads for key nodes across the gecko phylogeny estimated using Bayestraits over 5,000 trees from the Bayesian phylogenetic analysis. Numbers refer to node labels in panel B. B. Maximum likelihood tree showing phylogenetic relationships among gecko genera. The presence (red) or absence (black) of adhesive toepads is illustrated by colored squares on the tips of the branches (squares with two colors indicate polymorphism within the clade). Numbered nodes refer to Bayesian posterior distributions in panel A. C. Transition rate parameters from the Bayestraits analyses for the one rate model (in blue) and the two rate model where q01 =  gain of adhesive toepads (in red) and q10 =  loss of adhesive toepads (in black).
(PDF)

Figure S4 **Phylogenetic relationships among sampled gecko species and the evolution of adhesive toepads estimated using maximum likelihood.** Maximum likelihood tree showing phylogenetic relationships among sampled gecko species. Node color indicates ancestral states reconstructed using the mk1 model, summarized across a sample of 5,000 trees from the Bayesian phylogenetic analysis.
(PDF)

Figure S5 **Phylogenetic relationships among sampled gecko species and the evolution of adhesive toepads estimated using parsimony.** Maximum likelihood tree showing phylogenetic relationships among sampled gecko species. Node color indicates ancestral states reconstructed using parsimony (one of 114 equally parsimonious reconstructions).
(PDF)

Figure S6 **The number of transitions between the gain and loss of adhesive toepads in geckos.** Number of toepad gains (0 ->1) and losses (1 ->0) calculated using parsimony for 5,000 trees sampled from the Bayesian posterior distribution. Treescore  =  20.
(PDF)

Table S1 **Details of material examined.**
(PDF)

Table S2 **Summary of DNA sequence partitions.**
(PDF)
