# Supplementary figures and images for: Repeated Origin and Loss of Adhesive Toepads in Geckos
Source: PLoS One. 2012 Jun 27;7(6):e39429. doi: 10.1371/journal.pone.0039429 (PMC3384654; doi:10.1371/journal.pone.0039429)

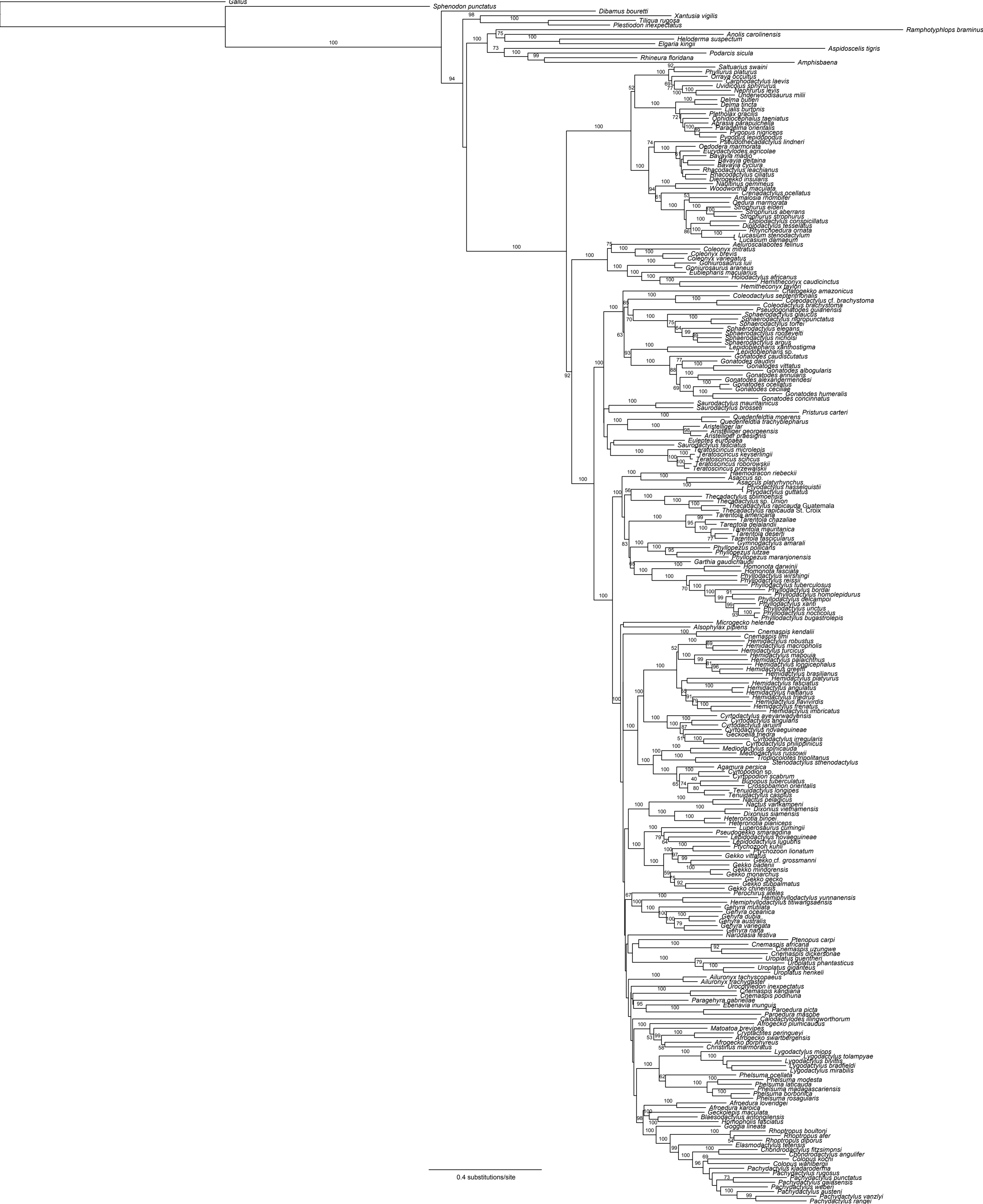

Supplement: Figure S1 — Phylogenetic relationships among sampled gecko species estimated using partitioned maximum likelihood. Bootstrap values from 100 rapid bootstrap replicates are shown at nodes. (PDF) [file pone.0039429.s001.pdf]

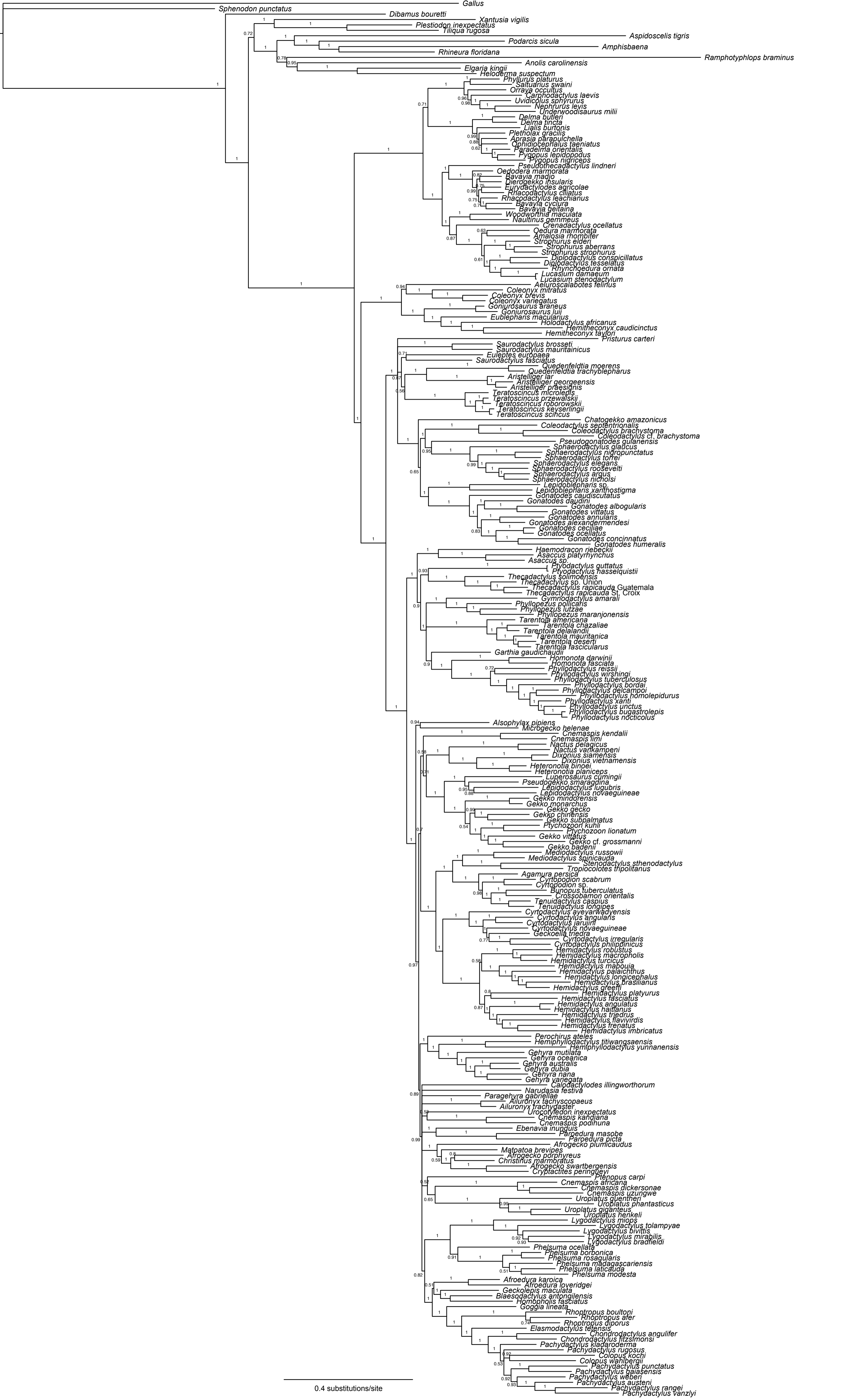

Supplement: Figure S2 — Phylogenetic relationships among sampled gecko species estimated using partitioned Bayesian analysis. Bayesian posterior probabilities are shown at nodes. (PDF) [file pone.0039429.s002.pdf]

**A**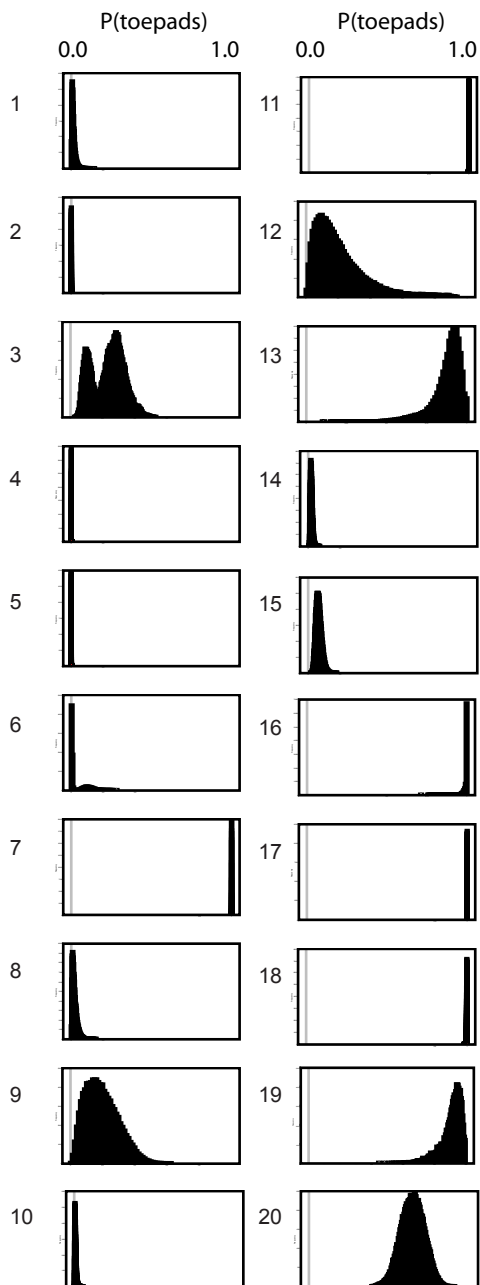**B**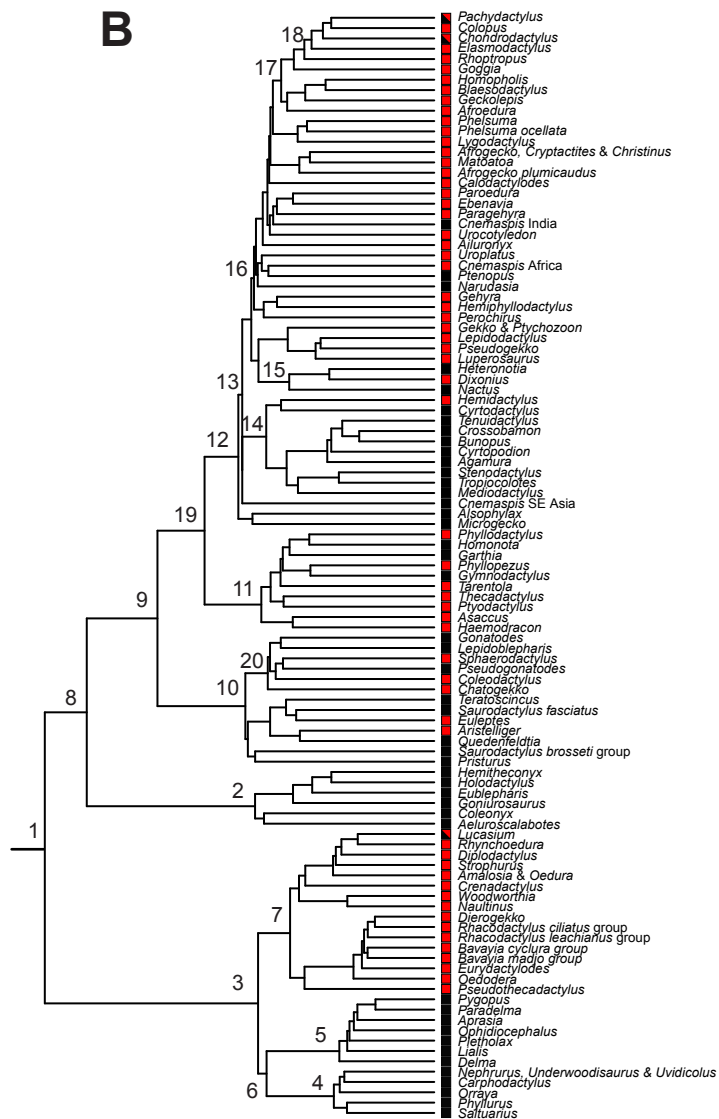**C**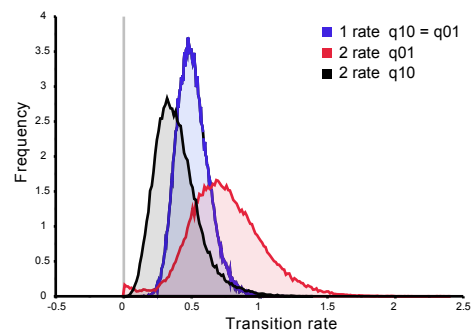

Supplement: Figure S3 — Gecko phylogeny and the evolution of adhesive toepads estimated using Bayesian methods. A. Bayesian posterior distributions of the presence of toepads for key nodes across the gecko phylogeny estimated using Bayestraits over 5,000 trees from the Bayesian phylogenetic analysis. Numbers refer to node labels in panel B. B. Maximum likelihood tree showing phylogenetic relationships among gecko genera. The presence (red) or absence (black) of adhesive toepads is illustrated by colored squares on the tips of the branches (squares with two colors indicate polymorphism within the clade). Numbered nodes refer to Bayesian posterior distributions in panel A. C. Transition rate parameters from the Bayestraits analyses for the one rate model (in blue) and the two rate model where q01 = gain of adhesive toepads (in red) and q10 = loss of adhesive toepads (in black). (PDF) [file pone.0039429.s003.pdf]

## Trace over Trees

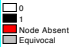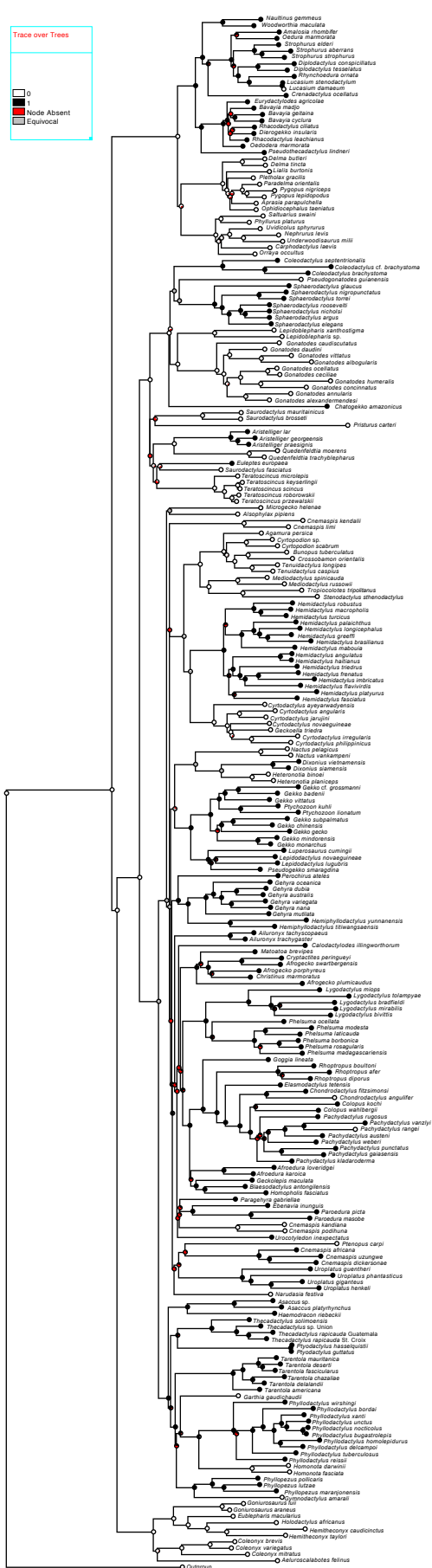

Supplement: Figure S4 — Phylogenetic relationships among sampled gecko species and the evolution of adhesive toepads estimated using maximum likelihood. Maximum likelihood tree showing phylogenetic relationships among sampled gecko species. Node color indicates ancestral states reconstructed using the mk1 model, summarized across a sample of 5,000 trees from the Bayesian phylogenetic analysis. (PDF) [file pone.0039429.s004.pdf]

☐ 0 (pads absent)  
☒ 1 (pads present)

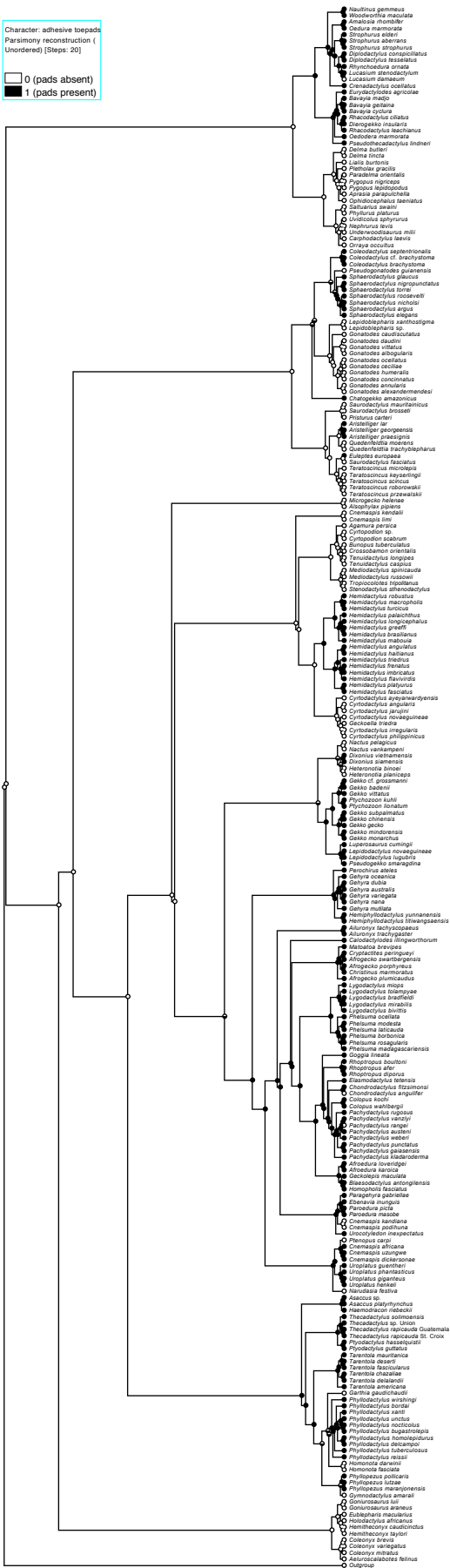

Supplement: Figure S5 — Phylogenetic relationships among sampled gecko species and the evolution of adhesive toepads estimated using parsimony. Maximum likelihood tree showing phylogenetic relationships among sampled gecko species. Node color indicates ancestral states reconstructed using parsimony (one of 114 equally parsimonious reconstructions). (PDF) [file pone.0039429.s005.pdf]

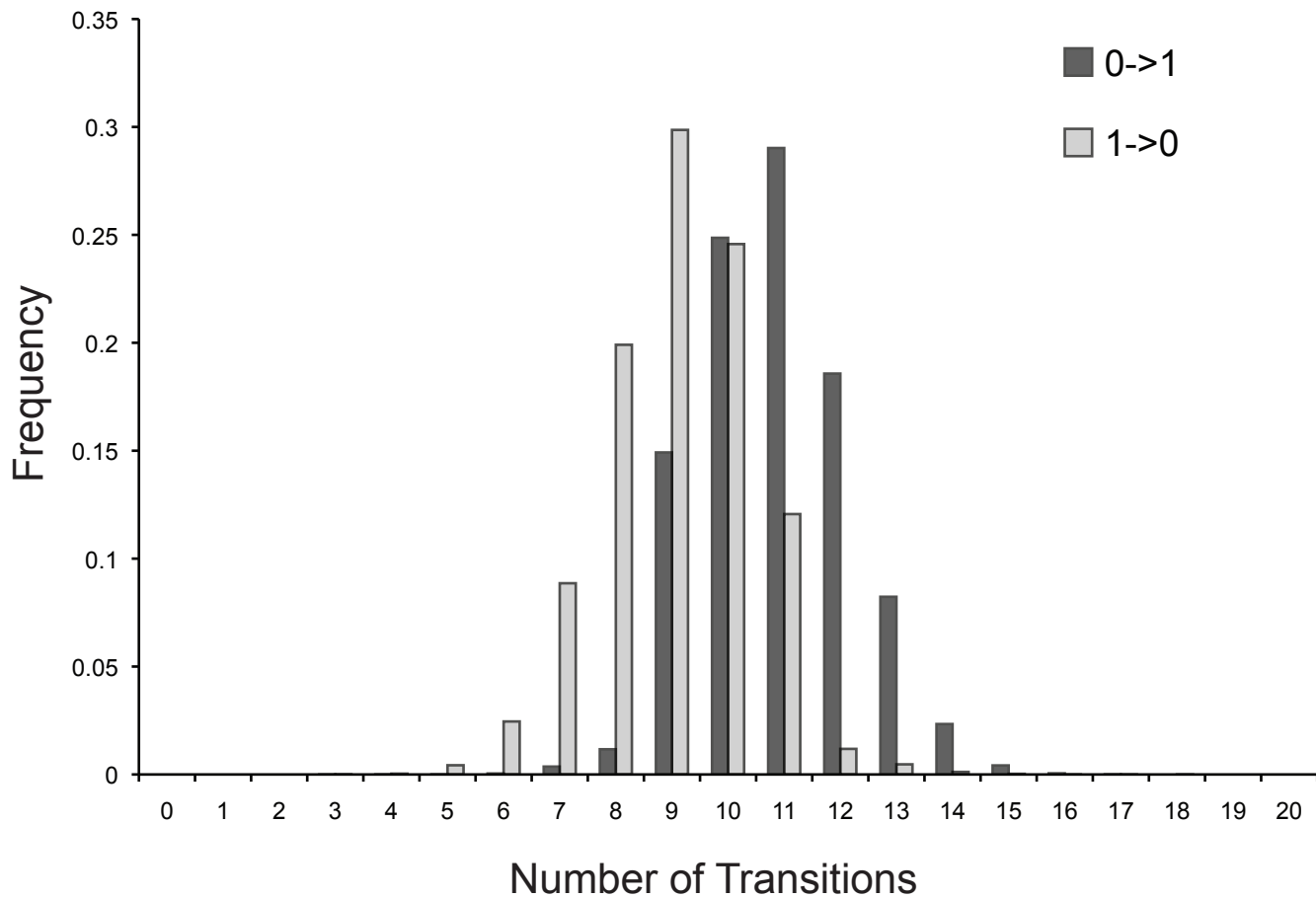

Supplement: Figure S6 — The number of transitions between the gain and loss of adhesive toepads in geckos. Number of toepad gains (0 ->1) and losses (1 ->0) calculated using parsimony for 5,000 trees sampled from the Bayesian posterior distribution. Treescore = 20. (PDF) [file pone.0039429.s006.pdf]
