# Supplementary material for: Repeated Origin and Loss of Adhesive Toepads in Geckos
Source: PLoS One. 2012 Jun 27;7(6):e39429. doi: 10.1371/journal.pone.0039429 (PMC3384654; doi:10.1371/journal.pone.0039429)
Supplement: Table S1 — Details of material examined. (PDF) [file pone.0039429.s007.pdf]

Table S1. Details of material examined. Family names abbreviated: E, Eublepharidae; D, Diplodactylidae; C, Carphodactylidae; Py, Pygopodidae; S, Sphaerodactylidae; Ph, Phyllodactylidae; G, Gekkonidae; and o, outgroups. Presence/absence of toepad morphologies are indicated. We used the following abbreviations: AMB, Aaron M. Bauer; AMCC, Ambrose Monell Cryo Collection, American Museum of Natural History; AMS, Australian Museum, Sydney; BPBM, Bernice P. Bishop Museum; BPN, Brice P. Noonan; CAS, California Academy of Sciences; CHUNB, Coleção Herpetológica da Universidade de Brasília; DB, Don Buden; ENS, Eric N. Smith; FG/MV, Frank Glaw and Miguel Vences; FGZC, Frank Glaw; FK, Fred Kraus; FLMNH, Florida Museum of Natural History; FMNH, Field Museum of Natural History; GVH, Gerald V Haagner; ID, Indraneil Das; JAC, Jonathan Campbell; JB, Jon Boone; JBL, Jonathan B. Losos - *Anolis* Genome Sequencing Project; JFBM, James Ford Bell Museum of Natural History, University of Minnesota; DJH, D. James Harris; JEM, John E. Measey; JS, Jay Sommers; JVV, Jens V. Vindum; KU, University of Kansas Museum of Natural History; LJAMM, Luciano J. Avila and Mariana Morando; LSHC, La Sierra University Herpetological Collection, L. Lee Grismer; LSUMZ, Louisiana State University Museum of Zoology; MCZ, Museum of Comparative Zoology, Harvard University; MF, Mike Forstner; MHNSM, Museo de Historia Natural, Universidad Nacional Mayor de San Marcos; MTSN, Trento Museum of Natural Sciences; MV, Museum of Victoria; MVZ, Museum of Vertebrate Zoology, Berkeley; MZUSP, Universidade de São Paulo, Museu de Zoologia; NMZ, National Museum of Zimbabwe; QM, Queensland Museum; PEM, Port Elizabeth Museum; RAH, Rod A. Hitchmough; RMB, Rafe M. Brown; ROM, Royal Ontario Museum; SAM, South Australian Museum; SC, Salvador Carranza; TG, Tony Gamble; WBJ, W. Bryan Jennings; WDH, Wulf D. Haacke; USNM, National Museum of Natural History, Smithsonian Institution; YPM, Yale Peabody Museum; ZCMV, Miguel Vences; ZFMK, Zoologisches Forschunginstitut und Museum Alexander Koenig; ZSM, Zoologische Staatssammlung München.

Family	Species	Toepads present	Leaf-toed morphology	Para-phalanges	ID	Locality	*RAG1*	*RAG2*	*C-MOS*	*ACM4*	*PDC*	*ND2* + tRNAs
C	*Carphodactylus laevis*	No	N/A	No	AMS 143258	Lamb Range, Queensland, Australia	EF534781	EF534947	EF534905	EF534862	EF534821	GU459943
C	*Nephrurus levis*	No	N/A	No	AMS 140561	Western Australia, Australia	GU459544	JQ945487	JQ945593	JQ945700	GU459746	AY369018
C	*Orraya occultus*	No	N/A	No	QM A002513	Queensland, Australia	JQ945320	–	–	–	JQ945388	JX041389
C	*Phyllurus platurus*	No	N/A	No	AMB 42	Sydney, NSW, Australia	HQ426314	HQ426488	HQ426570	HQ426397	HQ426226	JX024357
C	*Saltuarius swaini*	No	N/A	No	AMS 143262	Lamb Range, Queensland, Australia	JQ945338	JQ945509	JQ945616	JQ945722	JQ945407	JX024356
C	*Underwoodisaurus milii*	No	N/A	No	AMB 499	Denham, Western Australia, Australia	EF534780	EF534946	EF534904	EF534861	EF534820	JX041460
C	*Uvidicolus sphyrurus*	No	N/A	No	AMS R152351	Mt. Yulladunida, Kaputar Natl. Park, New South Wales, Australia	GU459543	JQ945521	JQ945627	JQ945734	GU459745	GU459944
D	*Amalosia rhombifer*	Yes	Yes	No	AMS 136216	vic. Bells Creek, Queensland, Australia	JQ945319	JQ945489	JQ945595	JQ945702	JQ945387	JX024363
D	*Bavayia cyclura*	Yes	No	No	AMB 7683	nr. Voh, New Caledonia	HQ426264	HQ426437	HQ426521	HQ426344	HQ426176	JX041315
D	*Bavayia geitaina*	Yes	No	No	AMB 7229	Mt. Ouin, New Caledonia	JQ945285	JQ945424	JQ945532	JQ945638	JQ945353	JX041316

Family	Species	Toepads present	Leaf-toed morphology	Para-phalanges	ID	Locality	*RAG1*	*RAG2*	*C-MOS*	*ACM4*	*PDC*	*ND2* + tRNAs
D	*Bavayia madjo*	Yes	No	No	AMS R149329	Mt. Panié, New Caledonia	JQ945286	JQ945425	JQ945533	JQ945639	JQ945354	GU459950
D	*Crenadactylus ocellatus*	Yes	Yes	No	AMS R162089	Trephina Gorge, Northern Territory, Australia	AY662627	JQ945439	FJ571641	JQ945652	JQ945367	JX024364
D	*Dierogekko insularis*	Yes	No	No	AMS R161069	Ile Art, Belep Ids., New Caledonia	JQ945306	JQ945448	JQ945555	JQ945661	JQ945375	JF972458
D	*Diplodactylus conspicillatus*	Yes	Yes	No	AMS 158426	Sturt Natl. Park, NSW, Australia	HQ426278	HQ426451	HQ426533	HQ426358	HQ426189	JQ173628
D	*Diplodactylus tesselatus*	Yes	Yes	No	AMS 143855	Stonehenge area, Queensland, Australia	JQ173725	JQ945449	JQ945556	JQ945662	JQ173677	JQ173631
D	*Eurydactylodes agricolae*	Yes	No	No	AMS R149366	Mt. Panié, New Caledonia	GU459547	JQ945453	JQ945560	JQ945666	GU459749	DQ533758
D	*Lucasium damaeum*	No	N/A	No	AMB 54	58 km S Alice Springs, NT, Australia	HQ426279	HQ426452	HQ426534	HQ426359	HQ426190	GU459953
D	*Lucasium stenodactylum*	Yes	Yes	No	AMS 139897	El Questro Station, Western Australia, Australia	JQ173724	JQ945477	JQ945584	JQ945690	JQ173676	JQ173630
D	*Naultinus gemmeus*	Yes	No	No	RAH 464	Hakataramea, New Zealand	GU459361	JQ945486	JQ945592	JQ945699	GU459563	GU459764
D	*Oedodera marmorata*	Yes	No	No	CAS 230936	Paagoumène, New Caledonia	JQ945318	JQ945488	JQ945594	JQ945701	JQ945386	GU459947
D	*Oedura marmorata*	Yes	Yes	No	AMS 143861	Stonehenge area, Queensland, Australia	EF534779	EF534945	EF534903	EF534860	EF534819	GU459951

Family	Species	Toepads present	Leaf-toed morphology	Para-phalanges	ID	Locality	*RAG1*	*RAG2*	*C-MOS*	*ACM4*	*PDC*	*ND2* + tRNAs
D	*Pseudothecadactylus lindneri*	Yes	No	No	MVZ 99544	Kakadu Natl. Park, NT, Australia	HQ426318	HQ426492	HQ426573	HQ426401	HQ426230	GU459946
D	*Rhacodactylus ciliatus*	Yes	No	No	TG 00080	New Caledonia	–	EF534944	EF534902	EF534859	–	–
D	*Rhacodactylus ciliatus*	Yes	No	No	AMS 146595	Rivière Bleue, New Caledonia	EF534778	–	–	–	EF534818	JX024438
D	*Rhacodactylus leachianus*	Yes	No	No	AMB 7189	Ilot Moro, New Caledonia	GU459548	JQ945505	JQ945612	JQ945718	GU459750	GU459949
D	*Rhynchoedura ornata*	Yes	Yes	No	AMS 155371	Sturt National Park, New South Wales, Australia	GU459553	JQ945508	JQ945615	JQ945721	GU459755	GU459954
D	*Strophurus aberrans*	Yes	Yes	No	AMS 136023	Tanami Road, Western Australia, Australia	JQ173761	JQ945511	–	JQ945724	JQ173715	JQ173667
D	*Strophurus elderi*	Yes	Yes	No	AMS 130987	Silver City Hwy, New South Wales, Australia	JQ173763	JQ945512	JQ945618	JQ945725	JQ173717	JQ173669
D	*Strophurus strophurus*	Yes	Yes	No	AMS 140536	Denham, Western Australia, Australia	JQ173766	JQ945513	JQ945619	JQ945726	JQ173720	JQ173672
D	*Woodworthia maculata*	Yes	No	No	RAH 292	Titahi Bay, New Zealand	GU459449	JQ945522	JQ945628	JQ945735	GU459651	GU459852
E	*Aeluroscalabotes felinus*	No	N/A	No	JB 16	Cameron Highlands, Malaysia	HQ426259	HQ426432	HQ426517	HQ426338	HQ426171	JX041301
E	*Coleonyx brevis*	No	N/A	No	TG 00194	Hudspeth County, Texas, USA	HQ426271	HQ426444	HQ426528	HQ426351	HQ426182	JX041333
E	*Coleonyx mitratus*	No	N/A	No	TG 00075	unknown	HQ426272	HQ426445	HQ426529	HQ426352	HQ426183	JX041334

Family	Species	Toepads present	Leaf-toed morphology	Para-phalanges	ID	Locality	*RAG1*	*RAG2*	*C-MOS*	*ACM4*	*PDC*	*ND2* + tRNAs
E	*Coleonyx variegatus*	No	N/A	No	CAS 205334	Imperial Co.,California, USA	EF534777	EF534943	EF534901	EF534858	EF534817	JX041335
E	*Eublepharis macularius*	No	N/A	No	JS 2	Pakistan	EF534776	–	–	–	EF534816	JX041350
E	*Eublepharis macularius*	No	N/A	No	TG 00081	Pakistan	–	EF534942	EF534900	EF534857	–	–
E	*Goniurosaurus araneus*	No	N/A	No	JFBM 15830	Vietnam	HQ426286	HQ426455	HQ426537	HQ426362	HQ426197	JX041364
E	*Goniurosaurus luii*	No	N/A	No	TG 00795	China	HQ426287	HQ426456	HQ426538	HQ426363	HQ426198	JX041365
E	*Hemitheconyx caudicinctus*	No	N/A	No	TG 00180	unknown	HQ426294	HQ426468	HQ426552	HQ426377	HQ426205	JX041370
E	*Hemitheconyx taylori*	No	N/A	No	JB 12	Somalia	HQ426295	HQ426469	HQ426553	HQ426378	HQ426206	JX041371
E	*Holodactylus africanus*	No	N/A	No	CAS 198845	Kajiado District, Kenya	HQ426296	HQ426470	HQ426554	HQ426379	HQ426207	JX041372
G	*Afroedura karroica*	Yes	Yes	Yes	PEM FN1112	Eastern Cape Province, South Africa	JQ945277	JQ945415	JQ945523	JQ945629	JQ945345	JX041302
G	*Afroedura loveridgei*	Yes	Yes	Yes	GVH 3969	Mozambique	JQ945278	JQ945416	JQ945524	JQ945630	JQ945346	JX041303
G	*Afrogecko plumicaudus*	Yes	Yes	No	WDH 1	Parque Nacional do Iona, Cunene Prov., Angola	JQ945279	JQ945417	JQ945525	JQ945631	JQ945347	JX041304
G	*Afrogecko porphyreus*	Yes	Yes	No	CAS 206995	Cape Hangklip, Western Cape Prov., South Africa	EF490723	JQ945418	JQ945526	JQ945632	EF490697	EF490776
G	*Afrogecko swartbergensis*	Yes	Yes	No	JB 47	Swartberg Mts., Western Cape Prov., South Africa	JQ945280	JQ945419	JQ945527	JQ945633	JQ945348	JX041305
G	*Agamura persica*	No	N/A	No	FMNH 247474	Makran Dist., Baluchistan,	JQ945281	JQ945420	JQ945528	JQ945634	JQ945349	JX041306

Family	Species	Toepads present	Leaf-toed morphology	Para-phalanges	ID	Locality	*RAG1*	*RAG2*	*C-MOS*	*ACM4*	*PDC*	*ND2* + tRNAs
						Pakistan						
G	*Ailuronyx tachyscopaeus*	Yes	No	No	MCZ F38717	Silhouette Island, Seychelles	JQ945282	JQ945421	JQ945529	JQ945635	JQ945350	JX041307
G	*Ailuronyx trachygaster*	Yes	No	No	AMB 8160	Silhouette Island, Seychelles	JQ945283	JQ945422	JQ945530	JQ945636	JQ945351	JX041308
G	*Alsophylax pipiens*	No	N/A	No	CAS 238805	Bulgan, Khovd, Mongolia	JQ945284	JQ945423	JQ945531	JQ945637	JQ945352	JX041309
G	*Blaesodactylus antongilensis*	Yes	No	Yes	ZSM 410/2005	Nosy Mangabe, Toamasina Prov., Madagascar	EU054229	JQ945426	JQ945534	JQ945640	EU054205	EU054253
G	*Bunopus tuberculatus*	No	N/A	No	CAS 228737	Sharjah, United Arab Emirates	JQ945287	JQ945427	JQ945535	JQ945641	JQ945355	JX041317
G	*Calodactylodes illingworthorum*	Yes	Yes	Yes	AMB7415	Serawa, Pitakumbura, Sri Lanka	JQ945288	JQ945428	JQ945536	JQ945642	JQ945356	JX041318
G	*Chondrodactylus angulifer*	No	N/A	No	MCZ R184984	Klein Aus Vista, Namibia	JQ945289	JQ945429	JQ945537	JQ945643	JQ945357	–
G	*Chondrodactylus angulifer*	No	N/A	No	AMB 4669	Richtersveld National Park, Northern Cape Prov., South Africa	–	–	–	–	–	JX041320
G	*Chondrodactylus fitzsimonsi*	Yes	No	No	CAS 193884	30 km N Swakopmund, Namibia	EU293645	EU293735	EU293690	EU293667	EU293712	JX041321
G	*Christinus marmoratus*	Yes	Yes	No	AMS 135338	Wirralie, Ladysmith, New South Wales, Australia	JQ945290	JQ945430	JQ945538	JQ945644	JQ945358	JX041322

Family	Species	Toepads present	Leaf-toed morphology	Para-phalanges	ID	Locality	*RAG1*	*RAG2*	*C-MOS*	*ACM4*	*PDC*	*ND2* + tRNAs
G	*Cnemaspis africana*	Yes	No	No	FMNH 251355	Amani, Tanga, Tanzania	–	JQ945431	JQ945539	JQ945645	–	–
G	*Cnemaspis africana*	Yes	No	No	CAS 168872	Amani, Tanga, Tanzania	JQ945291	–	–	–	JQ945359	JX041323
G	*Cnemaspis dickersonae*	Yes	No	No	MTSN 8604	Uzungwa Scarp, Tanzania	JQ945292	JQ945432	JQ945540	JQ945646	JQ945360	JX041324
G	*Cnemaspis kandiana*	No	N/A	No	AMB 7508	Masimbula, Godakawela, Sri Lanka	JQ945293	JQ945433	JQ945541	JQ945647	JQ945361	JX041325
G	*Cnemaspis kendalii*	No	N/A	No	LSHC 6562	Kepong, Selangor, Malaysia	JQ945294	JQ945434	JQ945542	–	JQ945362	JX041326
G	*Cnemaspis limi*	No	N/A	No	LSHC 6267	Pulau Tioman, Malaysia	EF534809	EF534977	EF534935	EF534892	EF534851	JX041327
G	*Cnemaspis podihuna*	No	N/A	No	AMB 7449	Mihintale, Sri Lanka	JQ945295	JQ945435	JQ945543	JQ945648	JQ945363	JX041328
G	*Cnemaspis uzungwe*	Yes	No	No	MTSN 5603	Chita, Uzungwe Scarp, Tanzania	JQ945296	JQ945436	JQ945544	JQ945649	JQ945364	JX041329
G	*Colopus kochi*	Yes	No	No	CAS 214308	59 km N Swakopmund, Namibia	JQ945297	JQ945437	JQ945545	JQ945650	JQ945365	JX041336
G	*Colopus wahlbergii*	Yes	No	No	NMZ 16974	Kalamba Station, Kazungula Dist., Zambia	JQ945298	JQ945438	JQ945546	JQ945651	JQ945366	JX041337
G	*Crossobamon orientalis*	No	N/A	No	ID 7618	vic. Sam, Rajasthan, India	JQ945299	JQ945440	JQ945547	JQ945653	JQ945368	JX041338
G	*Cryptactites peringueyi*	Yes	Yes	No	CAS 186374	Krom River Estuary, Easter Cape Prov., South Africa	JQ945300	JQ945441	JQ945548	JQ945654	JQ945369	JX041339

Family	Species	Toepads present	Leaf-toed morphology	Para-phalanges	ID	Locality	*RAG1*	*RAG2*	*C-MOS*	*ACM4*	*PDC*	*ND2* + tRNAs
G	*Cyrtodactylus angularis*	No	N/A	No	FMNH 265815	Muang Sa Kaeo, Sa Kaeo, Thailand	JQ945301	JQ945442	JQ945549	JQ945655	JQ945370	JX041340
G	*Cyrtodactylus ayeyarwadyensis*	No	N/A	No	CAS 216446	Rakhine, Myanmar	EU268287	JQ945443	JQ945550	JQ945656	EU268317	EU268348
G	*Cyrtodactylus irregularis*	No	N/A	No	FMNH 258697	Pakxong Dist., Champasak Prov., Lao PDR	JQ945302	JQ945444	JQ945551	JQ945657	JQ945371	JX041341
G	*Cyrtodactylus jarujini*	No	N/A	No	FMNH 255472	Thaphabat Dist., Bolikhamxay Prov., Lao PDR	JQ945303	JQ945445	JQ945552	JQ945658	JQ945372	JX041342
G	*Cyrtodactylus novaeguineae*	No	N/A	No	FK 11689	West Sepik, Papua New Guinea	HQ426274	HQ426447	HQ426531	HQ426354	HQ426185	JX041343
G	*Cyrtodactylus philippinicus*	No	N/A	No	FMNH 236073	Mt. Guitinguitin, Sibuyan Island, Philippines	JQ945304	JQ945446	JQ945553	JQ945659	JQ945373	JX041344
G	*Cyrtopodion scabrum*	No	N/A	No	TG 00109	Egypt	HQ426275	HQ426448	HQ426532	HQ426355	HQ426186	JX041345
G	*Cyrtopodion* sp.	No	N/A	No	FMNH 256409	Karakar Pass, Swat Dist., NW Frontier Prov., Pakistan	JQ945305	JQ945447	JQ945554	JQ945660	JQ945374	JX041346
G	*Dixonius siamensis*	No	N/A	No	LSHC 7328	Phnom Aural, Pursat Prov., Cambodia	EU054283	JQ945450	JQ945557	JQ945663	EU054267	EU054299
G	*Dixonius vietnamensis*	No	N/A	No	FMNH 263003	Keo Seima Dist., Mondolkiri Prov., Cambonia	EU054281	JQ945451	JQ945558	JQ945664	EU054265	EU054297

Family	Species	Toepads present	Leaf-toed morphology	Para-phalanges	ID	Locality	*RAG1*	*RAG2*	*C-MOS*	*ACM4*	*PDC*	*ND2* + tRNAs
G	*Ebenavia inunguis*	Yes	Yes	No	ZCMV 2099	Marojejy, Madagascar	HQ426280	HQ426453	HQ426535	HQ426360	HQ426191	JX041348
G	*Elasmodactylus tetensis*	Yes	No	No	PEM 5551	Niassa Game Reserve, Mozambique	JQ945307	JQ945452	JQ945559	JQ945665	JQ945376	JX041349
G	*Geckoella triedra*	No	N/A	No	35A	Sri Lanka	JQ945308	JQ945454	JQ945561	JQ945667	JQ945377	JX041352
G	*Geckolepis maculata*	Yes	No	Yes	FGZC 463	Montagne d'Ambre, Madagascar	EU054211	JQ945455	JQ945562	JQ945668	EU054187	EU054235
G	*Gehyra australis*	Yes	No	Yes	AMS 139934	El Questro Station, Western Australia, Australia	JN019145	JQ945456	JQ945563	JQ945669	JN019113	JN019081
G	*Gehyra* cf. *oceanica*	Yes	No	Yes	BPBM 23349	Parkop, Toricelli Mts., West Sepik Prov., Papua New Guinea	JQ945309	JQ945457	JQ945564	JQ945670	JN394000	JN393922
G	*Gehyra dubia*	Yes	No	Yes	AMS 152245	Daydawn, New South Wales, Australia	JN393956	JQ945458	JQ945565	JQ945671	JN393989	JN393911
G	*Gehyra mutilata*	Yes	No	Yes	JFBM 15819	unknown	–	JQ945459	JQ945566	JQ945672	–	–
G	*Gehyra mutilata*	Yes	No	Yes	AMB 6582	Penang, Malaysia	JN393962	–	–	–	JN393995	JN393917
G	*Gehyra nana*	Yes	No	Yes	AMS 140070	McGowens Beach, Kalumburu area, Western Australia, Australia	JN393963	JQ945460	JQ945567	JQ945673	JN393996	JN393918

**Family**	**Species**	**Toepads present**	**Leaf-toed morphology**	**Para-phalanges**	**ID**	**Locality**	***RAG1***	***RAG2***	***C-MOS***	***ACM4***	***PDC***	***ND2* + tRNAs**
G	*Gehyra variegata*	Yes	No	Yes	AMS 140478	Millstream, Western Australia, Australia	JN393973	JQ945461	JQ945568	JQ945674	JN394007	JN393929
G	*Gekko badenii*	Yes	No	No	TG 00095	Vietnam	–	JQ945462	JQ945569	JQ945675	–	–
G	*Gekko badenii*	Yes	No	No	JB 13	Vietnam	JN019130	–	–	–	JN019099	JN019065
G	*Gekko* cf. *grossmanni*	Yes	No	No	No ID	unknown	JN019129	JQ945463	JQ945570	JQ945676	JN019098	JN019064
G	*Gekko chinensis*	Yes	No	No	LSHC 4209	Wuzhi Shan, Hainan Id., China	JN019123	JQ945464	JQ945571	JQ945677	JN019092	JN019058
G	*Gekko gecko*	Yes	No	No	No ID	unknown	EF534813	–	–	–	EF534854	EU054288
G	*Gekko gecko*	Yes	No	No	TG 00079	Indonesia	–	EF534981	EF534939	EF534896	–	–
G	*Gekko mindorensis*	Yes	No	No	KU 303912	Barangay Formon, Sitio Balogbob, Cueba Simbahan, Mindoro Oriental Prov., Philippines	JN019140	JQ945465	JQ945572	JQ945678	JN019108	JN019076
G	*Gekko monarchus*	Yes	No	No	PEM R5412	Port Elizabeth, Eastern Cape, South Africa	JN019141	JQ945466	JQ945573	JQ945679	JN019109	JN019077
G	*Gekko subpalmatus*	Yes	No	No	AMB 6567	Chengdu, Sichuan, China	JN019128	JQ945467	JQ945574	JQ945680	JN019097	JN019063
G	*Gekko vittatus*	Yes	No	No	BPBM 19780	Rossel Id., Louisiade Ids., Milne Bay Prov., Papua New Guinea	JN019134	JQ945468	JQ945575	JQ945681	JN019102	JN019069

Family	Species	Toepads present	Leaf-toed morphology	Para-phalanges	ID	Locality	*RAG1*	*RAG2*	*C-MOS*	*ACM4*	*PDC*	*ND2* + tRNAs
G	*Goggia lineata*	Yes	Yes	No	AMB4762	Richtersveld National Park, Northern Cape Prov., South Africa	JQ945310	JQ945469	JQ945576	JQ945682	JQ945378	JX041353
G	*Hemidactylus angulatus*	Yes	No	Yes	MVZ 245438	Togo Hills, Nkwanta, Ghana	EU268306	–	HQ426540	HQ426365	EU268336	EU268367
G	*Hemidactylus brasilianus*	Yes	No	Yes	MZUSP 92493	Parque Nacional da Serra das Confusões, Piauí, Brazil	EU268290	HQ426439	HQ426523	HQ426346	EU268320	EU268351
G	*Hemidactylus fasciatus*	Yes	No	Yes	ROM 19891	Sapo Nat'l Park, Sinoe, Liberia	JQ945311	JQ945470	JQ945577	JQ945683	JQ945379	EU268371
G	*Hemidactylus flavivirdis*	Yes	No	Yes	FMNH 245515	Punjab Province, Pakistan	EU268294	HQ426458	HQ426541	HQ426366	EU268324	EU268355
G	*Hemidactylus frenatus*	Yes	No	Yes	TG 00088	Indonesia	–	EF534982	EF534940	EF534897	–	–
G	*Hemidactylus frenatus*	Yes	No	Yes	AMB 7411	Pidenipitiya, Sri Lanka	EF534814	–	–	–	EF534855	EU268357
G	*Hemidactylus greeffi*	Yes	No	Yes	CAS 219044	Praia da Mutamba, Sao Tomé	EU268308	HQ426459	HQ426542	HQ426367	EU268338	EU268369
G	*Hemidactylus haitianus*	Yes	No	Yes	CAS 198443	nr. Santo Domingo, Dominican Republic	HM559700	–	HQ426543	HQ426368	HM559667	HM559634
G	*Hemidactylus imbricatus*	Yes	No	Yes	TG 00568	Pakistan	HM559703	HQ426506	HQ426587	HQ426416	HM559670	EU268354
G	*Hemidactylus longicephalus*	Yes	No	Yes	CAS 218939	Lagoa Azul, Sao Tomé	HQ426289	HQ426460	HQ426544	HQ426369	HQ426200	HM559637
G	*Hemidactylus*	Yes	No	Yes	JEM 1864	Wundanyi,	HQ426291	HQ426462	HQ426546	HQ426371	HQ426202	JX041368

Family	Species	Toepads present	Leaf-toed morphology	Para-phalanges	ID	Locality	*RAG1*	*RAG2*	*C-MOS*	*ACM4*	*PDC*	*ND2* + tRNAs
	mabouia					Kenya						
G	*Hemidactylus macropholis*	Yes	No	Yes	CAS 227520	Bari Region, Puntland State, Somalia	HQ426292	HQ426463	HQ426547	HQ426372	HQ426203	JX041369
G	*Hemidactylus palaichthus*	Yes	No	Yes	LSUMZ H–12421	Roraima, Brazil	EU268307	HQ426464	HQ426548	HQ426373	EU268337	EU268368
G	*Hemidactylus platyurus*	Yes	No	Yes	JFBM 15815	unknown	HQ426273	HQ426446	HQ426530	HQ426353	HQ426184	–
G	*Hemidactylus platyurus*	Yes	No	Yes	KU30411	Lubang, Philippines	–	–	–	–	–	EU268352
G	*Hemidactylus robustus*	Yes	No	Yes	FMNH 245519	Makran District, Baluchistan Province, Pakistan	EU054271	HQ426465	HQ426549	HQ426374	EU054255	EU054287
G	*Hemidactylus triedrus*	Yes	No	Yes	JB 09	India	HM559715	HQ426466	HQ426550	HQ426375	HM559682	HM559649
G	*Hemidactylus turcicus*	Yes	No	Yes	TG 00567	Gainesville, Alachua County, Florida, USA	HQ426293	HQ426467	HQ426551	HQ426376	HQ426204	–
G	*Hemidactylus turcicus*	Yes	No	Yes	LSUMZ 1981	Baton Rouge, Louisiana, USA	–	–	–	–	–	EU268360
G	*Hemiphyllodactylus titiwangsaensis*	Yes	No	Yes	LSHC 7208	Cameron Highlands, Pahang, Malaysia	JN393978	JQ945471	JQ945578	JQ945684	JN394012	JN393934
G	*Hemiphyllodactylus yunnanensis*	Yes	No	Yes	FMNH 258695	Pakxong Dist., Champasak Prov., Lao PDR	JN393979	JQ945472	JQ945579	JQ945685	JN394013	JN393935
G	*Heteronotia binoei*	No	N/A	No	AMS 151170	Sturt Natl. Park, New South Wales,	EU054285	JQ945473	JQ945580	JQ945686	EU054269	EU054301

Family	Species	Toepads present	Leaf-toed morphology	Para-phalanges	ID	Locality	*RAG1*	*RAG2*	*C-MOS*	*ACM4*	*PDC*	*ND2* + tRNAs
						Australia						
G	*Heteronotia planiceps*	No	N/A	No	AMS 140331	23.3 km NNW jct. Tunnel Creek RD. with Great Northern Hwy., Western Australia, Australia	EU054284	JQ945474	JQ945581	JQ945687	EU054268	EU054300
G	*Homopholis fasciatus*	Yes	No	Yes	TG 00191	unknown	EU054226	JQ945475	JQ945582	JQ945688	EU054202	EU054250
G	*Lepidodactylus lugubris*	Yes	No	No	AMB 4111	Kirimati, Kiribati	EF534812	EF534980	EF534938	EF534895	EF534853	JX041377
G	*Lepidodactylus novaeguineae*	Yes	No	No	BPBM 15842	Boiaboiawaga Id., Milne Bay Prov., Papua New Guinea	JQ945312	JQ945476	JQ945583	JQ945689	JQ945380	JX041378
G	*Luperosaurus cumingii*	Yes	No	No	RMB 3546	Cumiagi, Philippines	JQ945313	JQ945478	JQ945585	JQ945691	JQ945381	JX041379
G	*Lygodactylus bivittis*	Yes	No	Yes	FG/MV 2001.A21	Andasibe, Madagascar	JQ945314	JQ945479	JQ945586	JQ945692	JQ945382	JX041380
G	*Lygodactylus bradfieldi*	Yes	No	Yes	AMB 7628	63.5 km W Kamanjab, Kunene Region, Namibia	HQ426301	HQ426474	HQ426558	HQ426383	HQ426212	JX041381
G	*Lygodactylus miops*	Yes	No	Yes	ZSM 116/2004	Andohahela, Madagascar	HQ426299	HQ426472	HQ426556	HQ426381	HQ426210	–
G	*Lygodactylus mirabilis*	Yes	No	Yes	FG/MV 2000.B3	Madagascar	HQ426300	HQ426473	HQ426557	HQ426382	HQ426211	JX041382
G	*Lygodactylus tolampyae*	Yes	No	Yes	FG/MV 2001.C14	Ankarafantsika, Madagascar	HQ426302	HQ426475	HQ426559	HQ426384	HQ426213	JX041383

Family	Species	Toepads present	Leaf-toed morphology	Para-phalanges	ID	Locality	*RAG1*	*RAG2*	*C-MOS*	*ACM4*	*PDC*	*ND2* + tRNAs
G	*Matoatoa brevipes*	Yes	Yes	No	FG/MV 2002.2237	Tulear area, Madagascar	EF490724	JQ945480	JQ945587	JQ945693	EF490698	EF490777
G	*Mediodactylus russowii*	No	N/A	No	AMB 8701	unknown	JQ945315	JQ945481	JQ945588	JQ945694	JQ945383	JX041384
G	*Mediodactylus spinicauda*	No	N/A	No	CAS 228709	Birjand, Khorasan Prov., Iran	JQ945316	JQ945482	JQ945589	JQ945695	JQ945384	JX041385
G	*Microgecko helenae*	No	N/A	No	JB 27	unknown	JQ945317	JQ945483	JQ945590	JQ945696	JQ945385	JX041386
G	*Nactus pelagicus*	No	N/A	No	CAS 229289	Mt. Gouémba, New Caledonia	EU054275	JQ945484	–	JQ945697	EU054259	EU054291
G	*Nactus vankampeni*	No	N/A	No	FK11384	Wewak, East Sepik Prov., Papua New Guinea	EU054279	JQ945485	JQ945591	JQ945698	EU054263	EU054295
G	*Narudasia festiva*	No	N/A	No	AMB 3243	Narudas, Namibia	EF534808	EF534976	EF534934	EF534891	EF534850	JX041387
G	*Pachydactylus austeni*	Yes	No	No	AMB 3295	Port Nolloth, Northern Cape Prov., South Africa	JQ945321	–	JQ945596	JQ945703	JQ945389	JX041390
G	*Pachydactylus gaiasensis*	Yes	No	No	AMB 7596	Gai-As, Namibia	JQ945322	JQ945490	JQ945597	JQ945704	JQ945390	JX041391
G	*Pachydactylus kladaroderma*	Yes	No	No	PEM FN1253	Molteno Pass, Western Cape Prov., South Africa	JQ945323	JQ945491	JQ945598	JQ945705	JQ945391	JX041392
G	*Pachydactylus punctatus*	Yes	No	No	MCZ R184457	Farm Celine, Limpopo Prov., South Africa	EU293646	–	–	–	EU293713	JX041393
G	*Pachydactylus punctatus*	Yes	No	No	MCZ R184458	Farm Celine, Limpopo Prov., South Africa	–	EU293736	EU293691	EU293668	–	–
G	*Pachydactylus rangei*	Yes	No	Yes	MCZ R183725	Swakop River, Namibia	JQ945324	JQ945492	JQ945599	–	JQ945392	JX041394

Family	Species	Toepads present	Leaf-toed morphology	Para-phalanges	ID	Locality	*RAG1*	*RAG2*	*C-MOS*	*ACM4*	*PDC*	*ND2* + tRNAs
G	*Pachydactylus rugosus*	Yes	No	No	CAS 201905	Sendelingsdrif, Richtersveld National Park, Northern Cape Prov., South Africa	JQ945325	JQ945493	JQ945600	JQ945706	JQ945393	JX041395
G	*Pachydactylus vanzlyi*	Yes	No	Yes	JVV 1761	Munutum River, Namibia	JQ945326	JQ945494	JQ945601	JQ945707	JQ945394	JX041396
G	*Pachydactylus weberi*	Yes	No	No	PEM R12449	1.4 km S Dawis, Richtersveld, Northern Cape Prov., South Africa	JQ945327	JQ945495	JQ945602	JQ945708	JQ945395	JX041397
G	*Paragehyra gabriellae*	Yes	No	No	FGZC 2366	Grotte Ampasy, Madagascar	JQ945328	JQ945496	JQ945603	JQ945709	JQ945396	JX041399
G	*Paroedura masobe*	Yes	Yes	No	JFBM 15832	Madagascar	EF536145	HQ426478	HQ426560	HQ426387	EF536169	EF536193
G	*Paroedura picta*	Yes	Yes	No	FG/MV 2002.B1	Berenty, Madagascar	EF536149	EU293737	EU293692	EU293669	EF536173	EF536197
G	*Perochirus ateles*	Yes	No	Yes	DB Dmale	Dehpelhi Id., Pohnpei, Federated States of Micronesia	JN393984	JQ945497	JQ945604	JQ945710	JN393946	JN393938
G	*Phelsuma borbonica*	Yes	No	No	JB 95	Réunion	HQ426305	HQ426479	HQ426561	HQ426388	HQ426216	JX041400
G	*Phelsuma laticauda*	Yes	No	No	TG 00232	Oahu, Hawaii	–	JQ945498	JQ945605	JQ945711	–	–
G	*Phelsuma laticauda*	Yes	No	No	FGZC 2705	Antalaha Airport, Madagascar	JQ945329	–	–	–	JQ945398	JX041401

Family	Species	Toepads present	Leaf-toed morphology	Para-phalanges	ID	Locality	*RAG1*	*RAG2*	*C-MOS*	*ACM4*	*PDC*	*ND2* + tRNAs
G	*Phelsuma madagascariensis*	Yes	No	No	FG/MV 2002.797	Manongarivo, Madagascar	EF534811	EF534979	EF534937	EF534894	AB081507	JX041402
G	*Phelsuma modesta*	Yes	No	No	ZSM 35/2004	Ambovombe, Madagascar	HQ426307	HQ426481	HQ426563	HQ426390	HQ426218	JX041403
G	*Phelsuma ocellata*	Yes	No	No	CAS 186351	22 km E Sendelingsdrif, Richtersveld National Park, Northern Cape, South Africa	HQ426308	HQ426482	HQ426564	HQ426391	HQ426219	JX041429
G	*Phelsuma rosagularis*	Yes	No	No	JB 109	Mauritius	HQ426306	HQ426480	HQ426562	HQ426389	HQ426217	JX041404
G	*Pseudogekko smaragdina*	Yes	No	No	KU 303995	Quezon, Philippines	JQ945332	JQ945501	JQ945608	JQ945714	JQ945401	JX041420
G	*Ptenopus carpi*	No	N/A	No	CAS 214534	20 km N Swakopmund, Namibia	JQ945333	JQ945502	JQ945609	JQ945715	JQ945402	JX041422
G	*Ptychozoon kuhli*	Yes	No	No	RMB 1134	Malaysia	JQ945334	JQ945503	JQ945610	JQ945716	JQ945403	JX041423
G	*Ptychozoon lionatum*	Yes	No	No	CAS 221168	Bago Div., Myanmar	JQ945335	JQ945504	JQ945611	JQ945717	JQ945404	JX041424
G	*Rhoptropus afer*	Yes	No	No	MCZ R183711	Rössing Mt., Namibia	JQ945336	JQ945506	JQ945613	JQ945719	JQ945405	JX041430
G	*Rhoptropus boultoni*	Yes	No	No	CAS 214713	Twyfelfontein, Namibia	EF534810	EF534978	EF534936	EF534893	EF534852	JX041431
G	*Rhoptropus diporus*	Yes	No	No	MCZ R183737	Brandberg Wes Myn, Namibia	JQ945337	–	–	–	JQ945406	JX041432
G	*Rhoptropus diporus*	Yes	No	No	MCZ R183736	Brandberg Wes Myn, Namibia	–	JQ945507	JQ945614	JQ945720	–	–
G	*Stenodactylus sthenodactylus*	No	N/A	No	MVZ 235804	Dakhlet Nouâdhibou Region, Mauritania	JQ945339	–	–	–	JQ945408	JX041441

Family	Species	Toepads present	Leaf-toed morphology	Para-phalanges	ID	Locality	*RAG1*	*RAG2*	*C-MOS*	*ACM4*	*PDC*	*ND2* + tRNAs
G	*Stenodactylus sthenodactylus*	No	N/A	No	TG 00181	Egypt	–	JQ945510	JQ945617	JQ945723	–	–
G	*Tenuidactylus caspius*	No	N/A	No	CAS 228602	Touran Protected Area, Semnan Prov., Iran	JQ945340	JQ945514	JQ945620	JQ945727	JQ945409	JX041448
G	*Tenuidactylus longipes*	No	N/A	No	CAS 228830	Tabas, Yazd Prov., Iran	JQ945341	JQ945515	JQ945621	JQ945728	JQ945410	JX041449
G	*Tropiocolotes tripolitanus*	No	N/A	No	FMNH 262276	Niger	–	JQ945517	JQ945623	JQ945730	–	–
G	*Tropiocolotes tripolitanus*	No	N/A	No	MVZ 238922	Tafokin, Agadez, Niger	JQ945343	–	–	–	JQ945412	JX041459
G	*Urocotyledon inexpectatus*	Yes	Yes	No	MCZF 38723	Silhouette Island, Seychelles	JQ945344	JQ945518	JQ945624	JQ945731	JQ945413	JX041461
G	*Uroplatus giganteus*	Yes	Yes	Yes	ZSM 55/2005	Marojejy, Madagascar	EF490737	JQ945519	JQ945625	JQ945732	JQ945414	EF490790
G	*Uroplatus guentheri*	Yes	Yes	Yes	ZSM 476/2001	Ankarafantsika, Madagascar	EF490725	JQ945520	JQ945626	JQ945733	EF490699	EF490778
G	*Uroplatus henkeli*	Yes	Yes	Yes	FG/MV 2000.C1	Nosy Be, Madagascar	EF490743	–	–	–	EF490716	EF490796
G	*Uroplatus henkeli*	Yes	Yes	Yes	JFBM 15833	Madagascar	–	HQ426510	HQ426591	HQ426420	–	–
G	*Uroplatus phantasticus*	Yes	Yes	Yes	FG/MV 2002.640	Ranomafana, Madagascar	EF490746	HQ426511	HQ426592	HQ426421	EF490719	EF490799
Ph	*Asaccus platyrhynchus*	Yes	Yes	No	CAS 227605	Wilayat Nazwa, Oman	EU293625	EU293715	EU293670	EU293647	EU293693	JX041313
Ph	*Asaccus sp.*	Yes	Yes	No	JB 15	Mirbat, Oman	EU293626	EU293716	EU293671	EU293648	EU293694	JX041314
Ph	*Garthia gaudichaudii*	No	N/A	No	SC 1	Chile	HQ426281	HQ426454	HQ426536	HQ426361	HQ426192	JX041351
Ph	*Gymnodactylus amarali*	No	N/A	No	CHUNB 38646	Cocalzinho, Goiás, Brazil	HQ426288	HQ426457	HQ426539	HQ426364	HQ426199	JX041366
Ph	*Haemodracon riebeckii*	Yes	Yes	No	JB 11	Socotra Island, Yemen	EU293627	EU293717	EU293672	EU293649	EU293695	JX041367
Ph	*Homonota darwinii*	No	N/A	No	LJAMM	Puerto	EU293628	EU293718	EU293673	EU293650	EU293696	JX041373

Family	Species	Toepads present	Leaf-toed morphology	Para-phalanges	ID	Locality	*RAG1*	*RAG2*	*C-MOS*	*ACM4*	*PDC*	*ND2* + tRNAs
					4601	Deseado, Santa Cruz, Argentina						
Ph	*Homonota fasciata*	No	N/A	No	TG 00085	Paraguay	EU293629	EU293719	EU293674	EU293651	EU293697	JX041374
Ph	*Phyllodactylus bordai*	Yes	Yes	No	AMCC 118242	Puebla, Mexico	HQ426309	HQ426483	HQ426565	HQ426392	HQ426220	JX041405
Ph	*Phyllodactylus bugastrolepis*	Yes	Yes	No	ROM 38489	Isla Santa Catalina, Baja California Sur, Mexico	EU293631	EU293721	EU293676	EU293653	EU293699	JX041406
Ph	*Phyllodactylus delcampoi*	Yes	Yes	No	JAC 21928	Guerrero, Mexico	HQ426310	HQ426484	HQ426566	HQ426393	HQ426221	JX041407
Ph	*Phyllodactylus homolepidurus*	Yes	Yes	No	MVZ 236267	Hermosillo, Sonora, Mexico	JQ945330	JQ945499	JQ945606	JQ945712	JQ945399	JX041408
Ph	*Phyllodactylus nocticolus*	Yes	Yes	No	ROM 39005	La Paz, Baja California Sur, Mexico	HQ426311	HQ426485	HQ426567	HQ426394	HQ426222	JX041409
Ph	*Phyllodactylus reissii*	Yes	Yes	No	JB 39	Peru	EU293632	EU293722	EU293677	EU293654	EU293700	JX041410
Ph	*Phyllodactylus tuberculosus*	Yes	Yes	No	KU 289758	PN El Imposible, Ahuachapán, El Salvador	EU293630	EU293720	EU293675	EU293652	EU293698	JX041411
Ph	*Phyllodactylus unctus*	Yes	Yes	No	ROM 39002	La Paz, Baja California Sur, Mexico	HQ426312	HQ426486	HQ426568	HQ426395	HQ426223	JX041412
Ph	*Phyllodactylus wirshingi*	Yes	Yes	No	TG 00722	Guanica, Puerto Rico	JQ945331	JQ945500	JQ945607	JQ945713	JQ945400	JX041413
Ph	*Phyllodactylus xanti*	Yes	Yes	No	ROM 38490	Baja California Sur, Mexico	EF534807	EF534975	EF534933	EF534890	EF534849	JX041414
Ph	*Phyllopezus lutzae*	Yes	No	Yes	CHUNB 50462	Mata de São João, Bahia, Brazil	HQ426265	HQ426438	HQ426522	HQ426345	HQ426177	JX041415

Family	Species	Toepads present	Leaf-toed morphology	Para-phalanges	ID	Locality	*RAG1*	*RAG2*	*C-MOS*	*ACM4*	*PDC*	*ND2* + tRNAs
Ph	*Phyllopezus maranjonensis*	Yes	No	Yes	ZFMK 84995	Balsas, Amazonas, Peru	EU293633	EU293723	EU293678	EU293655	EU293701	JX041416
Ph	*Phyllopezus pollicaris pollicaris*	Yes	No	Yes	MZUSP 92491	Parque Nacional da Serra das Confusões, Piauí, Brazil	EU293635	EU293725	EU293680	EU293657	EU293702	JX041417
Ph	*Ptyodactylus guttatus*	Yes	Yes	No	TG 00072	Egypt	EU293636	EU293726	EU293681	EU293658	EU293703	JX041425
Ph	*Ptyodactylus* cf. *hasselquistii*	Yes	Yes	No	YPM 13609	unknown	EU293637	EU293727	EU293682	EU293659	EU293704	JX041426
Ph	*Tarentola americana*	Yes	No	No	MVZ 241223	13 km E of Pilon, Granma Province, Cuba	HQ426332	HQ426503	HQ426584	HQ426413	HQ426243	JX041442
Ph	*Tarentola chazaliae*	Yes	No	No	TG 00130	Morocco	EU293638	EU293728	EU293683	EU293660	EU293705	JX041443
Ph	*Tarentola delalandii*	Yes	No	No	JB 43	Canary Islands	EU293639	EU293729	EU293684	EU293661	EU293706	JX041444
Ph	*Tarentola deserti*	Yes	No	No	JB 44	unknown	HQ426333	HQ426504	HQ426585	HQ426414	HQ426244	JX041445
Ph	*Tarentola fascicularus*	Yes	No	No	JB 29	unknown	HQ426334	HQ426505	HQ426586	HQ426415	HQ426245	JX041446
Ph	*Tarentola mauritanica*	Yes	No	No	TG 00129	Egypt	EU293641	EU293731	EU293686	EU293663	EU293708	JX041447
Ph	*Thecadactylus rapicauda*	Yes	No	Yes	USNM 561446	St. Croix, U.S. Virgin Islands	EU293643	EU293733	EU293688	EU293665	EU293710	JX041456
Ph	*Thecadactylus rapicauda*	Yes	No	Yes	ENS 7108	Izabal, Guatemala	EU293642	EU293732	EU293687	EU293664	EU293709	JX041455
Ph	*Thecadactylus solimoensis*	Yes	No	Yes	KU 214929	Cuzco Amazonico, Madre de Dios, Peru	EU293644	EU293734	EU293689	EU293666	EU293711	JX041457
Ph	*Thecadactylus* sp.	Yes	No	Yes	JB 25	Union Id., St. Vincent and Grenadines	JQ945342	JQ945516	JQ945622	JQ945729	JQ945411	JX041458

Family	Species	Toepads present	Leaf-toed morphology	Para-phalanges	ID	Locality	*RAG1*	*RAG2*	*C-MOS*	*ACM4*	*PDC*	*ND2* + tRNAs
Py	*Aprasia parapulchella*	No	N/A	No	MV D66569	Bendigo Whipstick, Victoria, Australia	HQ426260	HQ426433	AY134539	HQ426339	HQ426172	GU459941
Py	*Delma butleri*	No	N/A	No	SAM R36144	Coonbah, New South Wales, Australia	HQ426276	HQ426449	AY134548	HQ426356	HQ426187	AY134584
Py	*Delma tincta*	No	N/A	No	AMS 151607	Sturt Natl. Pk., NSW, Australia	HQ426277	HQ426450	AY172926	HQ426357	HQ426188	JX041347
Py	*Lialis burtonis*	No	N/A	No	TG 00078	Provinsi Papua, Indonesia	EF534782	EF534948	EF534906	EF534863	EF534822	JX024354
Py	*Ophidiocephalus taeniatus*	No	N/A	No	SAM R44653	Todmorden Station, South Australia, Australia	HQ426303	HQ426476	AY134565	HQ426385	HQ426214	AY134601
Py	*Paradelma orientalis*	No	N/A	No	QM J56089	20 km N Capella, Queensland, Australia	HQ426304	HQ426477	AY134569	HQ426386	HQ426215	AY134605
Py	*Pletholax gracilis*	No	N/A	No	WBJ 2483	Lesueur National Park, Western Australia, Australia	HQ426315	HQ426489	AY134566	HQ426398	HQ426227	AY134602
Py	*Pygopus lepidopodus*	No	N/A	No	WBJ 1206	Lesueur National Park, Western Australia, Australia	HQ426319	HQ426493	AY134567	HQ426402	HQ426231	AY134603
Py	*Pygopus nigriceps*	No	N/A	No	MVZ 197233	81 km S Alice Springs, Northern Territory, Australia	EF534783	EF534949	EF534907	EF534864	EF534823	JX024355

Family	Species	Toepads present	Leaf-toed morphology	Para-phalanges	ID	Locality	*RAG1*	*RAG2*	*C-MOS*	*ACM4*	*PDC*	*ND2* + tRNAs
S	*Aristelliger georgeensis*	Yes	No	No	JB 101	unknown	HQ426261	HQ426434	HQ426518	HQ426340	HQ426173	JX041310
S	*Aristelliger lar*	Yes	No	No	JB 01	Dominican Republic	EF534805	EF534973	EF534931	EF534888	EF534847	JX041311
S	*Aristelliger praesignis*	Yes	No	No	USNM 337563	Kingston, St. Andrew Parish, Jamaica	HQ426262	HQ426435	HQ426519	HQ426342	HQ426174	JX041312
S	*Chatogekko amazonicus*	Yes	Yes	No	LSUMZ H–16400	Manaus, Amazonas, Brazil	HQ426268	HQ426441	HQ426525	HQ426348	HQ426179	JX041319
S	*Coleodactylus brachystoma*	Yes	Yes	No	MZUSP 92569	Piauí, Brazil	EF534792	EF534959	EF534917	EF534874	EF534833	JX041330
S	*Coleodactylus cf. brachystoma*	Yes	Yes	No	CHUNB 43901	São Domingos, Goiás, Brazil	HQ426270	HQ426443	HQ426527	HQ426350	HQ426181	JX041331
S	*Coleodactylus septentrionalis*	Yes	Yes	No	LSUMZ H–12351	Roraima, Brazil	EF534791	EF534958	EF534916	EF534873	EF534832	JX041332
S	*Euleptes europaea*	Yes	Yes	No	No ID	Liguria, Italy	EF534806	EF534974	EF534932	EF534889	EF534848	JN393941
S	*Gonatodes albogularis*	No	N/A	No	MVZ 204073	Limon, Costa Rica	EF534797	–	–	–	EF534839	JX041354
S	*Gonatodes albogularis*	No	N/A	No	KU 289808	San Salvador, El Salvador	–	EF534965	EF534923	EF534880	–	–
S	*Gonatodes alexandermendesi*	No	N/A	No	BPN 1303	Imbaimadai, Guyana	EF534798	EF534966	EF534924	EF534881	EF534840	JX041355
S	*Gonatodes annularis*	No	N/A	No	ROM 22961	Guyana	–	EF534961	EF534919	EF534876	–	–
S	*Gonatodes annularis*	No	N/A	No	No ID	French Guiana	EF534794	–	–	–	EF534835	JX041356
S	*Gonatodes caudiscutatus*	No	N/A	No	KU 218359	Limon, Ecuador	EF534795	EF534962	EF534920	EF534877	EF534836	JX041357
S	*Gonatodes ceciliae*	No	N/A	No	TG 00039	Trinidad	–	EF564114	EF564088	EF564062	HQ426193	JX041358
S	*Gonatodes concinnatus*	No	N/A	No	LSUMZ H–12688	Sucumbios, Ecuador	HQ426282	EF564096	EF564070	EF564044	HQ426194	JX041359
S	*Gonatodes daudini*	No	N/A	No	JB 38	Union Id., St. Vincent and Grenadines	EF534793	EF534960	EF534918	EF534875	EF534834	JX041360
S	*Gonatodes humeralis*	No	N/A	No	MF 19492	Ecuador	EF534796	EF534964	EF534922	EF534879	EF534838	JX041361

Family	Species	Toepads present	Leaf-toed morphology	Para-phalanges	ID	Locality	*RAG1*	*RAG2*	*C-MOS*	*ACM4*	*PDC*	*ND2* + tRNAs
S	*Gonatodes ocellatus*	No	N/A	No	TG 00038	Tobago	HQ426284	EF564098	EF564072	EF564046	HQ426196	JX041362
S	*Gonatodes vittatus*	No	N/A	No	TG 00040	Trinidad	HQ426285	EF564112	EF564086	EF564060	–	JX041363
S	*Lepidoblepharis* sp.	No	N/A	No	KU 218367	Manabi, Ecuador	EF534789	EF534956	EF534914	EF534871	EF534830	JX041375
S	*Lepidoblepharis xanthostigma*	No	N/A	No	MVZ 171438	Limon, Costa Rica	EF534790	EF534957	EF534915	EF534872	EF534831	JX041376
S	*Pristurus carteri*	No	N/A	No	TG 00083	Yemen	EF534803	EF534971	EF534929	EF534886	EF534845	JX041419
S	*Pseudogonatodes guianensis*	No	N/A	No	KU 222142	Loreto, Peru	EF534784	EF534950	EF534908	EF534865	EF534824	JX041421
S	*Quedenfeldtia moerens*	No	N/A	No	JB 77	Moroccco	HQ426320	HQ426494	HQ426574	HQ426403	HQ426232	JX041427
S	*Quedenfeldtia trachyblepharus*	No	N/A	No	MVZ 178121	Oukaimeden, Morocco	EF534804	EF534972	EF534930	EF534887	EF534846	JX041428
S	*Saurodactylus brosseti*	No	N/A	No	TG 00082	Morocco	EF534802	EF534970	EF534928	EF534885	EF534844	JX041433
S	*Saurodactylus fasciatus*	No	N/A	No	DJH M616	Zumi, Morocco	HQ426322	HQ426495	HQ426576	HQ426405	HQ426234	JX041434
S	*Saurodactylus mauritanicus*	No	N/A	No	DJH Sm61	NW of Ain Benimather, Morocco	HQ426323	HQ426496	HQ426577	HQ426406	HQ426235	JX041435
S	*Sphaerodactylus argus*	Yes	Yes	No	TG 00125	Key West, Florida, USA	HQ426324	HQ426497	HQ426578	HQ426407	HQ426236	JX041436
S	*Sphaerodactylus elegans*	Yes	Yes	No	YPM 14795	Monroe County, Florida, USA	EF534787	EF534954	EF534912	EF534869	EF534828	JN393942
S	*Sphaerodactylus glaucus*	Yes	Yes	No	JAC 24229	Oaxaca, Mexico	HQ426325	HQ426498	HQ426579	HQ426408	HQ426237	JX041437
S	*Sphaerodactylus nicholsi*	Yes	Yes	No	TG 00211	Bahia de la Ballena, Puerto Rico	HQ426328	HQ426501	HQ426582	HQ426411	HQ426240	JX041438
S	*Sphaerodactylus nigropunctatus*	Yes	Yes	No	FLMNH 144010	Long Island, Bahamas	HQ426329	EF534953	EF534911	EF534868	EF534827	JX041439
S	*Sphaerodactylus roosevelti*	Yes	Yes	No	CAS 198428	Bahia de la Ballena, Puerto Rico	EF534785	EF534951	EF534909	EF534866	EF534825	JN393943

Family	Species	Toepads present	Leaf-toed morphology	Para-phalanges	ID	Locality	*RAG1*	*RAG2*	*C-MOS*	*ACM4*	*PDC*	*ND2* + tRNAs
S	*Sphaerodactylus torrei*	Yes	Yes	No	JB 34	Cuba	EF534788	EF534955	EF534913	EF534870	EF534829	JX041440
S	*Teratoscincus keyserlingii*	No	N/A	No	CAS 228808	Yazd Province, Iran	EF534801	HQ426508	HQ426589	HQ426418	EF534843	JX041450
S	*Teratoscincus microlepis*	No	N/A	No	TG 00074	Pakistan	EF534800	EF534968	EF534926	EF534883	EF534842	JX041451
S	*Teratoscincus przewalskii*	No	N/A	No	JFBM 15828	China	HQ426335	HQ426507	HQ426588	HQ426417	HQ426246	JX041452
S	*Teratoscincus roborowskii*	No	N/A	No	TG 00070	China	EF534799	EF534967	EF534925	EF534882	EF534841	JX041453
S	*Teratoscincus scincus*	No	N/A	No	JFBM 14252	Turkmenistan	HQ426336	EF534969	EF534927	EF534884	HQ426247	JX041454
o	*Amphisbaena alba*	No	N/A	No	CHUNB 38770	Distrito Federal, Brasil	AY662619	DQ119633	AY444016	HQ426422	HQ426249	AY662541
o	*Anolis carolinensis*	Yes	N/A	No	JBL SC1	South Carolina, USA	ENSACAT00000005087	ENSACAT00000005084	ENSACAG00000017993	ENSACAT00000004095	ENSACAT00000001314	–
0	*Anolis carolinensis*	Yes	N/A	No	JBL 982	Louisiana, USA	–	–	–	–	–	AF294279
o	*Aspidoscelis tigris*	No	N/A	No	TG 00069	Maricopa County, Arizona, USA	AY662620	HQ426512	AF039481	HQ426423	HQ426250	U71332
o	*Dibamus bouretti*	No	N/A	No	ROM 36056	Quang Thanh, Cao Bang, Vietnam	AY662645	HQ426513	AY662574	HQ426424	HQ426251	AY662562
o	*Elgaria kingii*	No	N/A	No	TG 00065	Navajo County, Arizona, USA	AY662603	HQ426514	AF039479	HQ426425	HQ426252	AF085618
o	*Gallus gallus*	No	N/A	No	-	-	NM001031188	AY443150	AY056925	NM001031191	XM426634	X52392
o	*Heloderma suspectum*	No	N/A	No	TG 00068	Arizona, USA	AY662606	DQ119635	AY662566	HQ426427	HQ426254	AB167711
o	*Plestiodon inexpectatus*	No	N/A	No	TG 00792	Florida, USA	AY662632	DQ119628	AY217888	HQ426426	HQ426253	AY607297
o	*Podarcis sicula*	No	N/A	No	TG 00124	Topeka, Kansas, USA	EF632239	DQ119630	EF679329	HQ426428	HQ426255	NC011609

Family	Species	Toepads present	Leaf-toed morphology	Para-phalanges	ID	Locality	*RAG1*	*RAG2*	*C-MOS*	*ACM4*	*PDC*	*ND2* + tRNAs
o	*Ramphotyphlops braminus*	No	N/A	No	No ID	Minneapolis, Minnesota, USA	AY662612	HQ426515	AF544717	HQ426429	HQ426256	AY662539
o	*Rhineura floridana*	No	N/A	No	FLMNH 141814	Alachua County, Florida, USA	AY662618	DQ119631	AY487347	EF534899	EU29371	AY605473
o	*Sphenodon punctatus*	No	N/A	No	No ID	New Zealand	AY662576	HQ426516	AF039483	HQ426430	HQ426257	AF534390
o	*Tiliqua rugosa*	No	N/A	No	JFBM 13685	New South Wales, Australia	EF534815	EF534983	EF534941	EF534898	EF534856	JX041462
o	*Xantusia vigilis*	No	N/A	No	TG 00121	Los Angeles County, California, USA	AY662642	DQ119626	AF148703	HQ426431	HQ426258	U71328
