# Supplementary material for: Repeated Origin and Loss of Adhesive Toepads in Geckos
Source: PLoS One. 2012 Jun 27;7(6):e39429. doi: 10.1371/journal.pone.0039429 (PMC3384654; doi:10.1371/journal.pone.0039429)
Supplement: Table S2 — Summary of DNA sequence partitions. (PDF) [file pone.0039429.s008.pdf]

Table S2. Summary of DNA sequence partitions. Estimated models of sequence evolution, total number of characters, number of variable sites and number of parsimony-informative sites for each data partition used in the phylogenetic analyses. The number of variable sites and parsimony-informative sites for just the Gekkota, excluding non-gekkotan outgroups, are also shown.

Partition	Model	Number of characters	Number of variable characters	Number of parsimony informative characters	Number of variable characters (Gekkota only)	Number of parsimony informative characters (Gekkota only)
nDNA codon 1	GTR+G	898	616	503	567	451
nDNA codon 2	GTR+I+G	897	533	417	478	360
nDNA codon 3	GTR+I+G	897	871	855	861	834
ND2 codon 1	GTR+I+G	334	310	289	308	287
ND2 codon 2	GTR+G	334	301	265	298	257
ND2 codon 3	GTR+I+G	334	334	334	334	334
tRNAs	GTR+I+G	412	385	351	382	346
